# In the diffuse large B-cell lymphoma microenvironment, SIRT1 is upregulated and correlated with a pro-inflammatory macrophage signature and autophagy-related gene expression

**DOI:** 10.3389/fimmu.2026.1701514

**Published:** 2026-02-04

**Authors:** Miguel Resanoa, Peio Azcoaga, Naike Salvador, Alba Delgado, Claudia Rodiño, Matthieu Schoenhals, Mounia S. Braza

**Affiliations:** 1 Department of Surgical Pathology, University Hospital of Navarra, Pamplona, Spain; 2 Biogipuzkoa Health Research Institute, San Sebastian, Spain; 3 Institute Pasteur of Madagascar, Antananarivo, Madagascar; 4 Department of Oncological Sciences, Icahn School of Medicine at Mount Sinai, New York, NY, United States; 5 Ikerbasque Basque Foundation for Science, Bilbao, Spain

**Keywords:** DLBCL - diffuse large B cell lymphoma, inflammation, macrophages, sirtuin (SIRT), tumor micreoenvironment (TME)

## Abstract

Diffuse large B-cell lymphoma (DLBCL) is an aggressive and heterogeneous blood cancer and one of the most frequent non-Hodgkin lymphomas of B-cell origin. As it has a complex, macrophage-rich immune microenvironment, we wanted to determine the role of autophagy, in which incoming threats are sequestered/removed and damaged cell constituents and debris are recycled, and metabolic sensors, such as sirtuins (SIRTs), in this cancer. Therefore, we determined the autophagy status in primary DLBCL samples using publicly available transcriptomic data and validated these results by immunohistochemistry and immunofluorescence analyses of patients’ tissue microarrays. We found that autophagy components and SIRTs were upregulated in the DLBCL microenvironment, particularly in the resting (M0) and pro-inflammatory (M1) macrophage subtypes. Moreover, the expression of autophagy factors was positively correlated with that of *SIRT1* and *SIRT3*, which were both upregulated in macrophages. Specifically, *SIRT1* was correlated with the expression of *CD80* (M1 macrophage marker) and *SIRT3* with the expression of *M-CSF* (M2 macrophage marker). Overall, in DLBCL samples, we observed a positive correlation between the expression of *SIRT1* and of inflammation-related genes, and between *SIRT3* and immunosuppression-related genes. Lastly, we confirmed in an independent DLBCL cohort that only SIRT1, but not SIRT3, was significantly associated with autophagy-related immune cells. Our study identified SIRT expression in macrophages of the DLBCL environment and specifically the importance of SIRT1 in the DLBCL M1 macrophage immune microenvironment. This opens an avenue for the potential translational exploitation of SIRT1 modulation as therapeutic target in this hematological malignancy.

## Introduction

Non-Hodgkin lymphoma describes a heterogeneous group of lymphoid malignancies that make up 90% of all hematological cancers. Lymphomagenesis is complex due to a biased immunological response (1) caused by the specific immune microenvironment with different immune cell populations that might become tumor-supportive when interacting with malignant B cells (2, 3). Diffuse large B-cell lymphoma (DLBCL) is an aggressive and genetically very complex lymphoid malignancy of B-cell origin. It is also a morphologically heterogeneous tumor with considerable variations in clinical course and response to therapy (1, 4–7). Due to its aggressive and highly proliferative behavior, DLBCL requires significant amounts of energy and metabolic precursors.

Sirtuins (SIRT) are conserved nicotinamide adenine dinucleotide (NAD^+^)-dependent lysine deacetylases involved in metabolism regulation. In mammals, there are seven sirtuins (SIRT1-SIRT7) that display various enzymatic activities, including lysine deacetylation, desuccinylation and defattyacylation. Some sirtuins have oncogenic functions while others have tumor-suppressive functions (8–11). Specifically, NAD^+^ depletion induces cell death in blood cancer cells, suggesting that sirtuins are crucial for their survival (12). In hematologic malignancies, SIRT1, SIRT3 and SIRT6 are implicated in mitochondrial and autophagy processes, and may have oncogenic or tumor-suppressive functions, depending on the tumor microenvironment status (13–18). SIRT1 has a crucial, but controversial role in cancer (19). It might serve as an oncogenic (20, 21) or as a tumor-suppressor protein (8). It has been mostly studied in childhood malignancies (e.g. acute lymphoblastic leukemia/lymphoma) (22, 23) and in chronic lymphocytic leukemia/small lymphocytic lymphoma (24, 25) in which its expression level is elevated compared with normal cells. SIRT3 is primarily located in mitochondria. Its role in the host defense has been analyzed in detail (26). Conversely, its implication in hematologic malignancies is controversial (13, 16, 18). Moreover, very little is known about SIRT1 and SIRT3 roles in autophagy and immune signatures in the DLBCL microenvironment.

Autophagy is a metabolic process in which unnecessary or dysfunctional components are degraded and recycled through the fusion of autophagosomes (double-membrane vesicles) with lysosomes (membrane-bound organelles). Autophagy is essential for damage clearance to maintain the cellular homeostasis (27–30). Growing evidence indicates that it also plays important and opposite roles in different cancer types and in their treatment outcome depending on their microenvironment (29, 31–33). For instance, autophagy can suppress tumorigenesis by removing damaged organelles/proteins and by limiting cell growth and genomic instability (32). On the other hand, induction of autophagy by metabolic stress in apoptosis-deficient tumor cells can support tumor cell survival (34). As metabolic functions, such as autophagy, play primordial roles in homeostasis in both physiological and pathological conditions (especially in the regulation of innate immune responses) (35–37), we hypothesized that SIRT1 and SIRT3 might be implicated in DLBCL metabolism and that this might have consequences on the innate immune system (e.g. macrophages).

Therefore, we investigated SIRT1 and SIRT3 expression profiles in DLBCL and its immune microenvironment (macrophages), and their link with autophagy using data from publicly available databases: The Cancer Genome Atlas (TCGA) for DLBCL samples and Genotype-Tissue Expression (GTEx) for control samples. We validated our bioinformatic results in an independent DLBCL sample cohort included in tissue microarrays (TMA). Our study shows that in DLBCL, SIRT1 and SIRT3 are specifically upregulated in macrophages and correlated with a segregated immune signature, and that SIRT1 is linked to a pro-autophagic profile.

## Materials and methods

For the bioinformatic analysis, we exploited a TCGA dataset of 48 DLBCL samples. However, only 47 of these samples were included in GEPIA2 (the patients’ characteristics are summarized in **Supplementary Table S1**). We also used 337 spleen control tissue samples from the GTEx database, which is the most important database in term of control cohorts. We selected spleen among the secondary lymphoid organs available in the GTEx control list because of its similarity to lymph nodes in term of immune cell composition and characteristics compared to the other available controls. In addition, we used an independent cohort of 192 samples in TMAs for the immunohistochemistry (IHC) analysis.

### Gene expression profiling interactive analysis

As previously described (38), we used the GEPIA server (39) and its new version (40) for gene expression profiling in non-tumor controls and DLBCL samples using data from publicly available mRNA sequencing databases (GTEx and TCGA, respectively). Each sample tool in TCGA/GTEx was deconvoluted with the CIBERSORT-ABS, EPIC and quanTIseq tools. Based on the inferred cell proportions in each bulk-RNA sample, we performed several downstream analyses, including proportion, correlation, sub-expression, and survival analyses. We displayed the results as boxplots (gene expression), correlation graphs (correlation analyses) and histograms (deconvolution analyses).

### TIMER2.0

We analyzed the immune cell infiltrate with TIMER2.0 (*http://timer.cistrome.org/**)* using different algorithms, as previously described (41, 42). We performed a computational deconvolution with high-accuracy and well-defined cell-type signatures to estimate the tumor-infiltrating immune cell populations using different algorithms, such as CIBERSORT-ABS and EPIC. We also evaluated the tumor-infiltrating M1 and M2 macrophage subpopulations. We used the list of differentially expressed genes in DLBCL *versus* spleen samples to characterize changes in tumor-infiltrating immune cell populations.

### Tissue microarrays and immunohistochemistry

For the IHC analysis, we used commercially available TMAs (Tissuearray, LY2086b) that included 176 lymphoma samples of different subtypes (118 DLBCL, 3 Burkitt-like lymphoma, 5 follicular lymphoma, 1 mantle cell lymphoma, 4 plasma cell lymphoma, 7 anaplastic large cell lymphoma, 22 T-cell lymphoma, 4 angioimmunoblastic T-cell lymphoma, 12 Hodgkin’s lymphoma) and 16 lymph node samples as controls. Each TMA included one single core per sample. The patients’ characteristics are summarized in **Supplementary Table S2**. After heat-induced epitope retrieval, we incubated TMA sections with anti-CD80 (Novus Biologicals, AF140, USA), anti-M-CSF (Novus Biologicals, AF216, USA), anti-SIRT1 (Abcam, Ab110304, UK) and anti-SIRT3 (Abcam, Ab217319, UK) antibodies at 4 °C overnight, followed by incubation with biotin-streptavidin horseradish peroxidase-conjugated secondary antibodies and 3,3′ -diaminobenzidine. We quantified the percentage of positive cells per core with a light microscope (Nikon 80i). We calculated the histoscore of each sample as previously described (38).

### Tissue microarrays and immunofluorescence

For immunofluorescence (IF) staining, we used the same TMAs used for IHC staining. Briefly, we dried TMA sections in an oven at 73°C for 10 minutes. After dewaxing and rehydrating with decreasing concentrations of alcohol, we performed antigen recovery by incubating sections in Tris-EDTA buffer at pH9 in a microwave for 25 minutes. We blocked non-specific binding by incubating samples with 10% donkey serum for 1 hour. Then, we incubated sections with the primary antibodies at 4 °C overnight and with the secondary antibodies at room temperature for 1 hour. To eliminate autofluorescence, we incubated samples in Sudan Black for 1 hour. The primary antibodies were against SIRT1 and SIRT3 (ab110304 and ab217319, respectively, Abcam, UK), beclin-1 (ab114071, ab62557, Abcam, UK), CD20 (ab64088, Abcam, UK), CD68 (ab201340, Abcam, UK), CD86 (ab269587, Abcam, UK), CD206 (ab64693, Abcam, UK), CD3 (ab5690, Abcam, UK), CD80 and M-CSF (AF140, AF216, Novus, UK). For IF image analysis, we assessed the double and triple positive staining subsets by quantifying the percentage of cells in which different signals co-localized relative to all DAPI-positive cells.

In rare cases when specific antibodies could not be used together (a maximum of four- color staining was possible, DAPI included), we exploited the peri-tumor or intra-tumor localization and the immune cell characteristics (size and morphology) to correlate the IF staining to the cell type (macrophages and tumor cells). The analysis and results were validated by a pathologist (co-author in this manuscript).

We acquired images at 20X and 40X resolution with a Zeiss AxioObserver 7 microscope and ZEN, version 3.7. For the quantification of IF signals:

- For DAPI, we used the Dragonfly software, version 2022.1 (Comet Technologies Canada Inc., Montreal, Canada; software available at https://www.theobjects.com/dragonfly). We determined cell numbers in two steps: segmentation and counting. For the segmentation step, we trained an artificial intelligence model to distinguish two regions of interest: cells and background. Then, we applied this model to the section images. We removed spots smaller than 5µm to prevent considering small particles as cells.
- For the specific antibody signals, we quantified them manually in each TMA core (DLBCL and control) in triplicate (i.e., three repeated segmentations).

### Statistical analysis

We used fold change, ranks and correlation coefficients to describe the results. We considered significant p-values ≤0.05. For the pairwise gene expression correlation analysis using TCGA and GTEx data, we used the Pearson, Spearman and Kendall correlation coefficients. GEPIA uses the non-log scale for calculation and the log-scale axis for visualization. We used one-way ANOVA to compare differential gene expression, cell type proportions and sub-expression analyses using the sample status (DLBCL or Normal) as displayed by box plots. We estimated the survival contribution of specific autophagy- and inflammation-related genes expressed in DLBCL using the Mantel–Cox test that displays the results as log10 hazard ratios (HR). We presented the IHC and IF results as percentages ± SD and compared them with the 2-tailed unpaired Student’s *t*-test or the Mann-Whitney test.

The high p-values obtained in this study, despite modest visual differences in some cases, are due to the small sample size (e.g., 48 DLBCL samples from TCGA) and low variability in some datasets that increase statistical power.

## Results

### The expression levels of SIRT1, SIRT3 and several autophagy components are increased in the DLBCL microenvironment and are correlated with distinct macrophage subtypes

**Figure 1:**
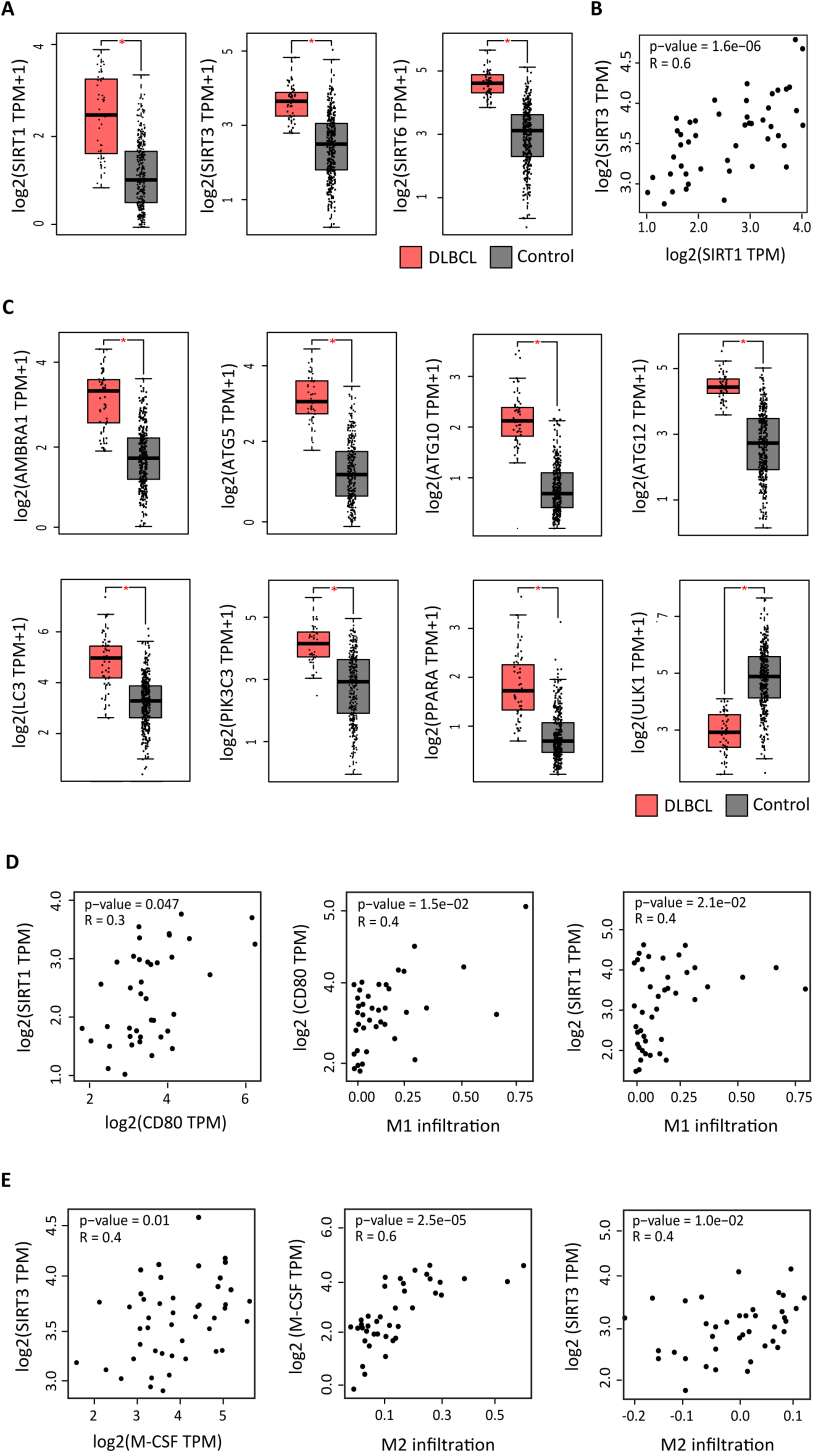
Expression of autophagy components in DLBCL and spleen (control) samples. Box plots showing the tissue-wide expression profiles of autophagy genes in 47 DLBCL samples (TCGA database) (red boxes) and 337 spleen tissue samples (GTEx database) (gray boxes). **(A)** Expression levels of the indicated autophagy factors (log2 (TPM + 1). **(B)***SIRT1*, *3* and *6* gene expression levels (log2 (TPM + 1). TPM: transcript count per million reads. **(C)** Correlation of *SIRT1* and *SIRT3* expression. **(D)** Correlation of *SIRT1* expression with *CD80* expression, of *CD80* expression with M1 macrophage infiltration and of *SIRT1* with M1 macrophage infiltration in DLBCL. **(E)** Correlation of *SIRT3* expression with *M-CSF* expression, of *M-CSF* expression with M1 macrophage infiltration and of *SIRT3* with M2 macrophage infiltration in DLBCL. Significance: P ≤0.05.

In a previous study, we identified a strong pro-inflammatory signal in the macrophage-rich DLBCL microenvironment (38), in line with the observations made by Kotlov et al. (43). Now, we wanted to know whether this pro-inflammatory signal was associated with metabolic deregulation. Therefore, first, we compared the gene expression profiles of 47 DLBCL samples from the TCGA database and 337 healthy spleen samples. Among the differentially expressed metabolic targets, we found that *SIRT1, SIRT3* and *SIRT6* were significantly upregulated in DLBCL samples compared with controls (P ≤0.05) (**Figure 1A**). Nevertheless, only the expression levels of *SIRT1* and *SIRT3* were significantly correlated (R = 0.64 p =1.6e-06) (**Figure 1B**). Although these genes were not the top overexpressed genes in our DLBCL cohort (**Supplementary Figure S1**), we chose them due to their relevance and key roles in many important metabolic processes in other cancers. Specifically, *SIRT1* and *SIRT3* are mechanistically linked to autophagy, metabolism, and immune regulation in cancer, and are consistently associated in hematologic malignancies (10). Moreover, pharmacological strategies targeting *SIRT2*, *SIRT6* and *SIRT7* are less advanced, limiting their immediate translational potential.

As some studies (8, 9, 26) suggested that sirtuins regulate autophagy, a process related to cell stress and contributing also to the cell metabolism, we asked whether SIRT1 and SIRT3 upregulation in DLBCL was accompanied by deregulation of autophagy components. Therefore, we investigated the expression profiles of autophagy-related genes in the DLBCL microenvironment and their potential link to SIRT1 and SIRT3. Most autophagy-related genes (*AMBRA1, ATG5, ATG10, ATG12, LC3B, PIK3C3, PPARA*) were significantly upregulated (P ≤0.05) except for *ULK1* that was significantly downregulated in tumors versus controls (**Figure 1C**) (44).

In parallel, we used the TIMER2.0 bioinformatics tool and the CIBERSORT-ABS and EPIC algorithms to investigate the DLBCL microenvironment infiltration by several immune cell subtypes: B cells, CD4^+^ T and CD8^+^ T cells, dendritic cells, macrophages (M0, M1 and M2), monocytes, natural killer cells, neutrophils, and regulatory T cells. Regardless of the bioinformatics tool and source, and in line with our previous results (38), the macrophage infiltration scores, especially for the M1 subpopulation, were significantly higher in DLBCL than in control samples and were higher than those of all other immune cells, except for B cells (**Supplementary Figures S2A-C**). We next examined the correlation between *SIRT1*, *SIRT3* and *SIRT6* expression and *CD80* and *M-CSF* (45, 46) (M1 and M2 macrophage marker, respectively) expression in DLBCL samples. The expression level of *SIRT1* was positively correlated with that of *CD80*, which was correlated with M1 macrophage infiltration level. Indeed, *SIRT1* expression levels were significantly correlated with *CD80* expression and infiltration by the M1 macrophage subpopulation (R = 0.29 p =0.047, R = 0.38; p = 1.53e-02 and R = 0.36 p = 2.1e-02), (**Figure 1D**). *SIRT3* expression level was positively correlated with that of *M-CSF*, which was correlated with M2 macrophage infiltration level. Consequently, *SIRT3* expression was significantly correlated with *M-CSF* expression and infiltration by the M2 macrophage subpopulation (R = 0.37 p =0.01, R = 0.61; p = 2.49e-05 and R = 0.40 p = 1.0e-02), (**Figure 1E**). Lastly, we investigated whether the expression levels of the genes of interest in our study (*CD68, CD80, M-CSF, SIRT1* and *SIRT3*) varied in DLBCL classified in different stages (I, II, II and IV), but we did not find any significant difference (**Supplementary Figure S3**).

Taken together, these data suggest that SIRT1 and SIRT3 may be expressed by specific macrophage populations.

### SIRT1 expression is correlated with the expression levels of autophagy and pro-inflammatory factors in DLBCL

**Figure 2:**
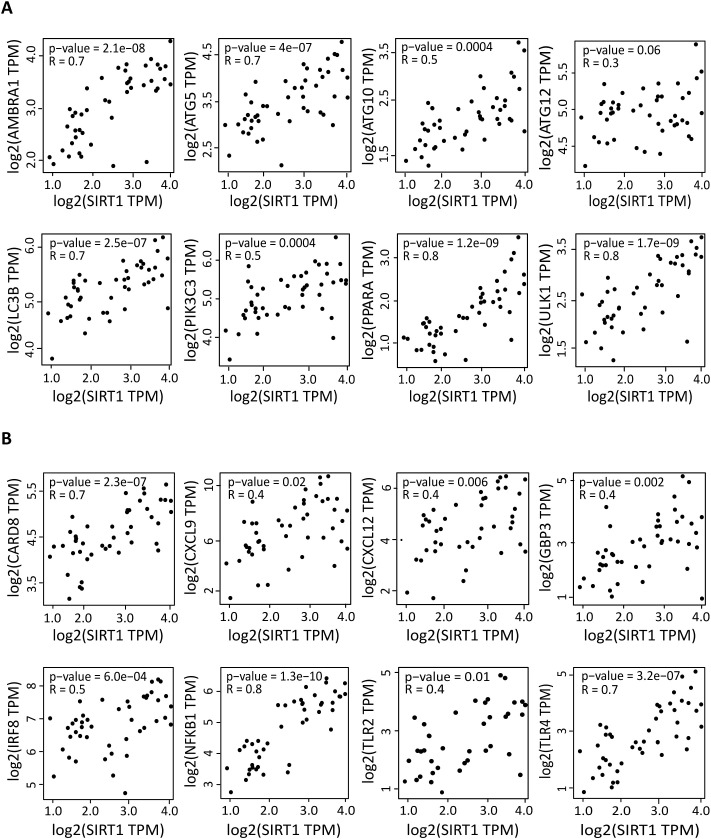
Correlation of the expression of autophagy and inflammation genes with *SIRT1* expression in DLBCL. Correlation between the gene expression levels of the indicated autophagy **(A)** and inflammation **(B)** factors and *SIRT1* in 47 DLBCL samples (TCGA database). Quantitative comparisons based on the Pearson correlation coefficient (R). Y-axis: log2 (TPM) of the indicated gene expression levels, X-axis: log2 (TPM) of *SIRT1* expression level. TPM, transcript count per million reads. Significance: P ≤0.05.

We next examined whether *SIRT1* expression was correlated with that of autophagy components in DLBCL. In DLBCL samples, *SIRT1* expression was positively correlated with the expression of several key autophagy markers (*AMBRA1, ATG5, ATG10, ATG12, LC3B, PIK3C3, PPARA* and *ULK1*) (0.31≤ R ≤0.89, 2.7e-12≤ p ≤0.032) (**Figure 2A**). Although *ULK1* was significantly downregulated in DLBCL samples compared with healthy spleen controls, its expression within the DLBCL cohort remained positively correlated with SIRT1. This could be explained by its variable expression in tumor samples. Indeed, ULK1 was globally downregulated in DLBCL samples, yet its relative variation among tumors could be still associated with SIRT1 levels, consistent with their known regulatory role in autophagy.

As *SIRT1* expression was correlated with that of the pro-inflammatory marker *CD80* and with M1 macrophage infiltration, we wondered whether *SIRT1* was linked to inflammation in DLBCL. *SIRT1* expression was positively correlated with that of genes implicated in the inflammatory response (*CARD8, CXCL9, CXCL11, CXCL12, GBP3, IRF8, NFKB1, TLR2* and *TLR4*: 0.25≤ R ≤0.78; 1.3e-10≤ p ≤0.018) (**Figure 2B**) (38). Altogether, these results indicated a strong correlation between *SIRT1* expression, autophagy and inflammatory components in DLBCL.

### SIRT3 expression is correlated with the expression levels of autophagy and anti-inflammatory factors in DLBCL

**Figure 3:**
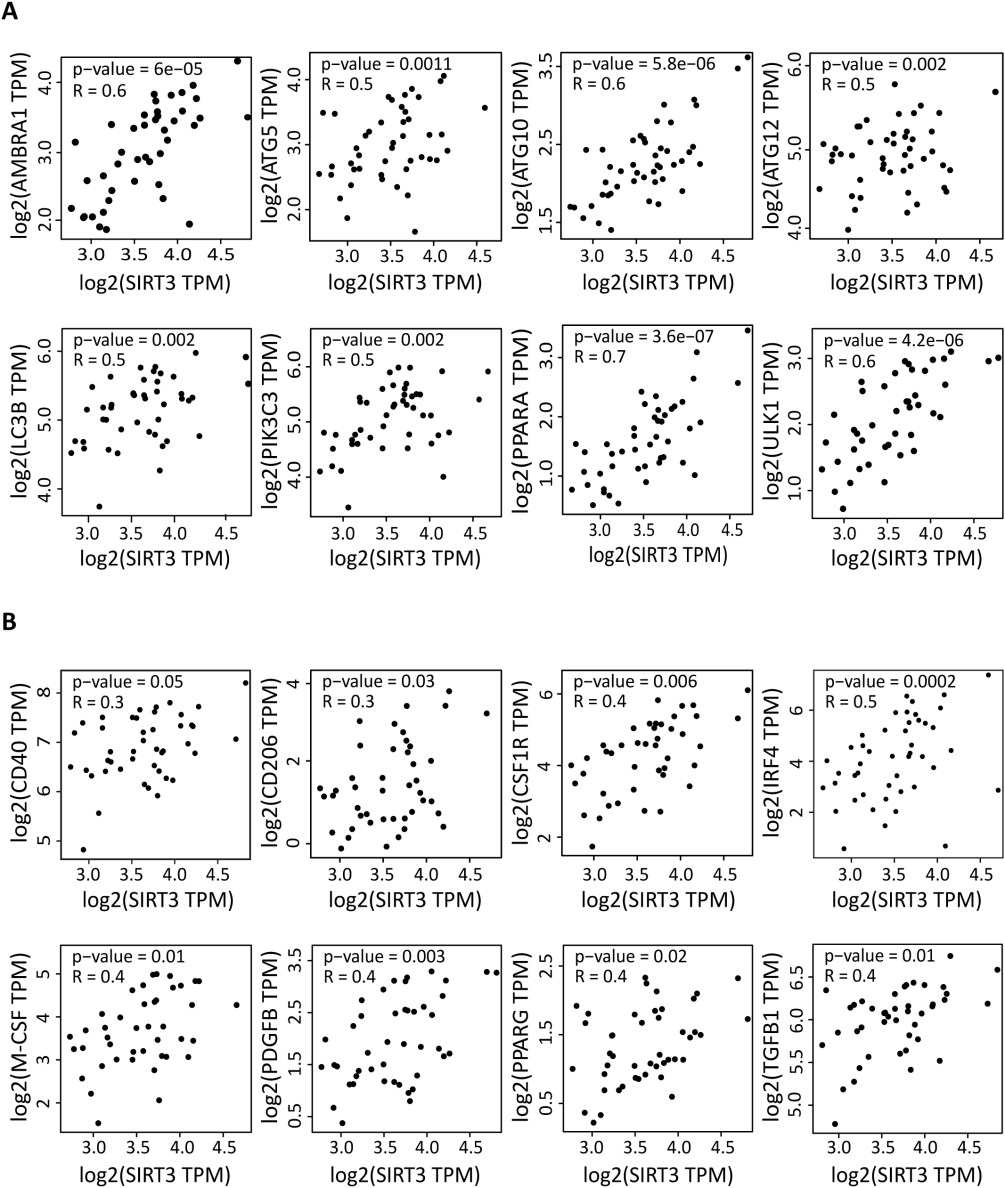
Correlation of the expression of autophagy and inflammation genes with *SIRT3* expression in DLBCL. Correlation between the gene expression levels of the indicated autophagy **(A)** and inflammation **(B)** factors and *SIRT3* in 47 DLBCL samples (TCGA database). Quantitative comparisons based on the Pearson correlation coefficient (R). Y-axis: log2 (TPM) of the indicated gene expression levels, X-axis: log2 (TPM) of *SIRT3* expression level. TPM, transcript count per million reads. Significance: P ≤0.05.

In DLBCL samples, *SIRT3* expression was positively correlated with the expression of several key autophagy markers (*AMBRA1, ATG5, ATG10, ATG12, LC3B, PIK3C3, PPARA* and *ULK1*) (0.44≤ R ≤0.66, 3.6e-07≤ p ≤0.0018) (**Figure 3A**). As *SIRT3* expression was correlated with that of the anti-inflammatory marker *M-CSF* and with M2 macrophage infiltration, we asked whether *SIRT3* was linked to immunosuppression in DLBCL. *SIRT3* expression was positively correlated (**Figure 3B**) with that of the anti-inflammatory genes *CD206, CD40, CSF1R, PDGFB, PPARG* and *TGFB1* (0.29≤ R ≤0.43; 0.0026≤ p ≤0.048) (**Figure 3B**) (47). Altogether, these results indicated a strong correlation of *SIRT3* expression with autophagy and anti-inflammatory components in DLBCL.

### The expression levels of autophagy components and of SIRT1 and SIRT3 are increased in M0 and M1 macrophages in the DLBCL microenvironment, accompanied with an enrichment in mTOR expression associated with SIRT1

**Figure 4:**
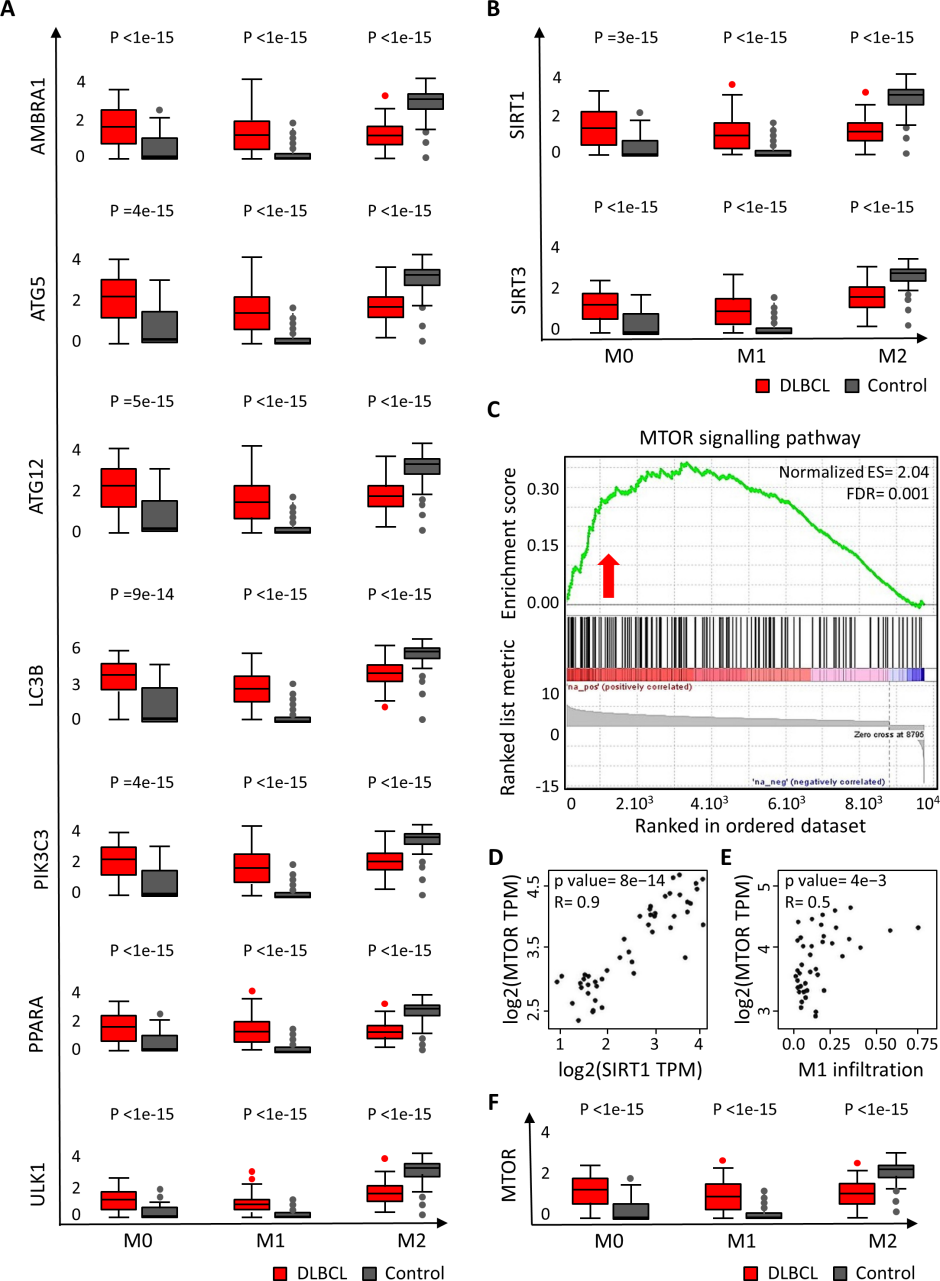
Differential expression of autophagy and metabolic components in M0, M1 and M2 macrophages in DLBCL and their link with the mTOR signaling pathway. **(A)** Comparison of the gene expression of key autophagy factors in M0, M1 and M2 macrophages in 47 DLBCL samples (TCGA database) and 337 spleen tissue samples (normal secondary lymphoid organ tissue; GTEx database). Y-axis: log (TPM + 1) of the gene expression levels. TPM: transcript count per million reads. The figure shows the results of the deconvolution analyses according to the tissue type: DLBCL (red) and control (gray). Significance: P ≤0.05 (one-way ANOVA). **(B)** Comparison of *SIRT1* and *SIRT3* expression in M0, M1 and M2 macrophages in 47 DLBCL samples (TCGA database) and 337 spleen tissue samples (normal secondary lymphoid organ tissue; GTEx database). Y-axis: log (TPM + 1) of the gene expression levels. TPM: transcript count per million reads. The figure shows the results of the deconvolution analyses according to the tissue type: DLBCL (red) and control (gray). Significance: P ≤0.05 (one-way ANOVA). **(C)** Gene set enrichment analysis (GSEA) plots for the mammalian target of rapamycin (mTOR) pathway using the raw DLBCL and control transcriptome datasets. The red to blue horizontal bar represents the ranked list. Red arrows indicate upregulation of the mTOR pathway in the pairwise comparisons. FDR: False discovery rate. Significance: P ≤0.05. **(D)** Correlation between the gene expression levels of *SIRT1* and *MTOR* in 47 DLBCL samples (TCGA database). Quantitative comparisons based on the Pearson correlation coefficient (R). Y-axis: log2 (TPM) of the *MTOR* gene expression levels, X-axis: log2 (TPM) of *SIRT1* expression level. TPM: transcript count per million reads. Significance: P ≤0.05. **(E)** Correlation between *MTOR* expression and M1 macrophage subtype infiltration levels in 47 DLBCL samples (TCGA database). Quantitative comparisons based on the Pearson correlation coefficient (R). Y-axis: log2 (TPM) of the *MTOR* gene expression levels, X-axis: log2 (TPM) of M1 macrophages infiltration level. TPM: transcript count per million reads. Significance: P ≤0.05. **(F)** Comparison of *MTOR* expression in M0, M1 and M2 macrophages in 47 DLBCL samples (TCGA database) and 337 spleen tissue samples (normal secondary lymphoid organ tissue; GTEx database). Y-axis: log (TPM + 1) of the gene expression levels. TPM, transcript count per million reads. The figure shows the results of the deconvolution analyses according to the tissue type: DLBCL (red) and control (gray). Significance: P ≤0.05 (one-way ANOVA).

The findings in the previous sections showed an increased expression of autophagy markers in the DLBCL microenvironment that was significantly correlated with the upregulation of *SIRT1* and *SIRT3* (associated with pro-inflammatory and anti-inflammatory gene signatures, respectively). Therefore, using deconvolution analysis (**Supplementary Figure S4**), we investigated the expression of autophagy factors in M0, M1 and M2 macrophages in DLBCL samples and in controls. Key autophagy factors (*AMBRA1*, *ATG5*, *ATG12*, *LC3B*, *PIK3C3*, *PPARA*, *SIRT1*, *SIRT3* and *ULK1*) were significantly upregulated in M0 and M1 macrophages in DLBCL compared with control samples (tumor/control fold change: 1.4e+01 to 5.1e+04, p ≤1.0e-14 for M0; 2.9e+02 to 2.7e+06, p ≤1.0e-15 for M1, respectively), but not in M2 macrophages (tumor/control fold change: 4.1e-04 to 8.7e-01, p ≤1.e-15) (**Figures 4A, B**, **Table 1**). In line with our previous results, this suggests that in DLBCL, key autophagy components, together with SIRT1 and SIRT3, are mainly induced in M0 and M1 macrophages. Then, we investigated the different dysregulated pathways and their enrichment in the studied DLBCL cohort compared with controls. We found a significant enrichment in the mTOR signaling pathway (ES = 2.04, FDR = 0.001) that is frequently involved in cancers as a pro-tumor factor (**Figure 4C**). As the mTOR pathway is enriched in the DLBCL microenvironment, we examined whether mTOR was correlated with M1 or M2 macrophage infiltration and with the expression of SIRT1 or SIRT3. We found that *MTOR* expression was significantly and positively correlated with *SIRT1* expression (R = 0.85, p= 8e-14) (**Figure 4D**), with M1 macrophage infiltration (R = 0.45, p = 4e-3) (**Figure 4E**), and with *BECN1* (gene encoding beclin-1) expression (R = 0.76, p = 4e-10) (data not shown). Moreover, by assessing the expression of *MTOR* in M0, M1 and M2 macrophages in DLBCL samples and in controls, we found that it was significantly upregulated in M0 and M1 macrophages (tumor/control fold change: 4.6e+01, p ≤1.0e-15 for M0; 7.1e+02, p ≤1.0e-15 for M1, respectively) and significantly downregulated in M2 macrophages (tumor/control fold change: 4.6e-01, p ≤1.e-15) (**Figure 4F**). In line with our previous results, this suggests that in the DLBCL microenvironment, and especially in the macrophage compartment, the mTOR signaling pathway is deregulated.

**Table 1:** Differentially expressed autophagy components in the three macrophage subtypes in DLBCL.

Genes	M0		M1		M2	
	Tumor/control	P value	Tumor/control	P value	Tumor/control	P value
AMBRA1	5.1e+04	<1e-15	8.0e+02	<1e-15	4.5e-04	<1e-15
ATG5	1.4e+04	=4e-15	2.7e+06	<1e-15	6.7e-01	<1e-15
ATG12	3.0e+04	=5e-15	1.5e+06	<1e-15	5.7e-01	<1e-15
LC3B	1.4e+01	09e-14	2.2e+03	<1e-15	8.7e-01	<1e-15
PIK3C3	3.1e+04	=4e-15	1.4e+06	<1e-15	5.6e-01	<1e-15
SIRT1	3.3e+04	=3e-15	1.3e+06	<1e-15	6.2e-01	<1e-15
SIRT3	5.6e+01	<1e-15	5.5e+02	<1e-15	4.5e-01	<1e-15
PPARA	5.6e+01	<1e-15	6.4e+02	<1e-15	4.1e-04	<1e-15
ULK1	7.0e+01	<1e-15	2.9e+02	<1e-15	4.5e-04	<1e-15

Tumor/control fold change of gene expression [Log(TPM + 1)] of autophagy components in M0, M1 and M2 macrophages in DLBCL samples and control samples, and p values. E: Exponential function. P ≤0.05 was considered significant.

Then, we wanted to determine whether the survival of patients with DLBCL was influenced by the expression levels of these autophagy and metabolic factors. Using the Mantel–Cox test (cutoff-low: 50%, cutoff-high: 50%), we performed a Kaplan Meier and Log Rank analysis (survival contribution of each individual gene) and survival maps (survival contribution of different genes). We found that the expression level of the tested autophagy and metabolic factors did not affect survival, likely due to the limited sample size (**Supplementary Figure S5**). Overall, our results show that autophagy components and the *SIRT1*/*SIRT3* metabolic sensors are highly expressed in the M0 and M1 macrophage sub-populations and that the mTOR signature is biased in our DLBCL cohort.

### SIRT1 and SIRT3 expression levels are correlated with those of CD80 and M-CSF, respectively, in the DLBCL microenvironment

To confirm some of the results obtained with the bioinformatics analysis in independent samples, we performed IHC using TMAs that contained, in total, 118 DLBCL tumors and 16 normal lymphoid tissue samples (appendix, bone marrow, lymph nodes, placenta, spleen, thymus, and tonsil) (**Supplementary Table S2**).

**Figure 5:**
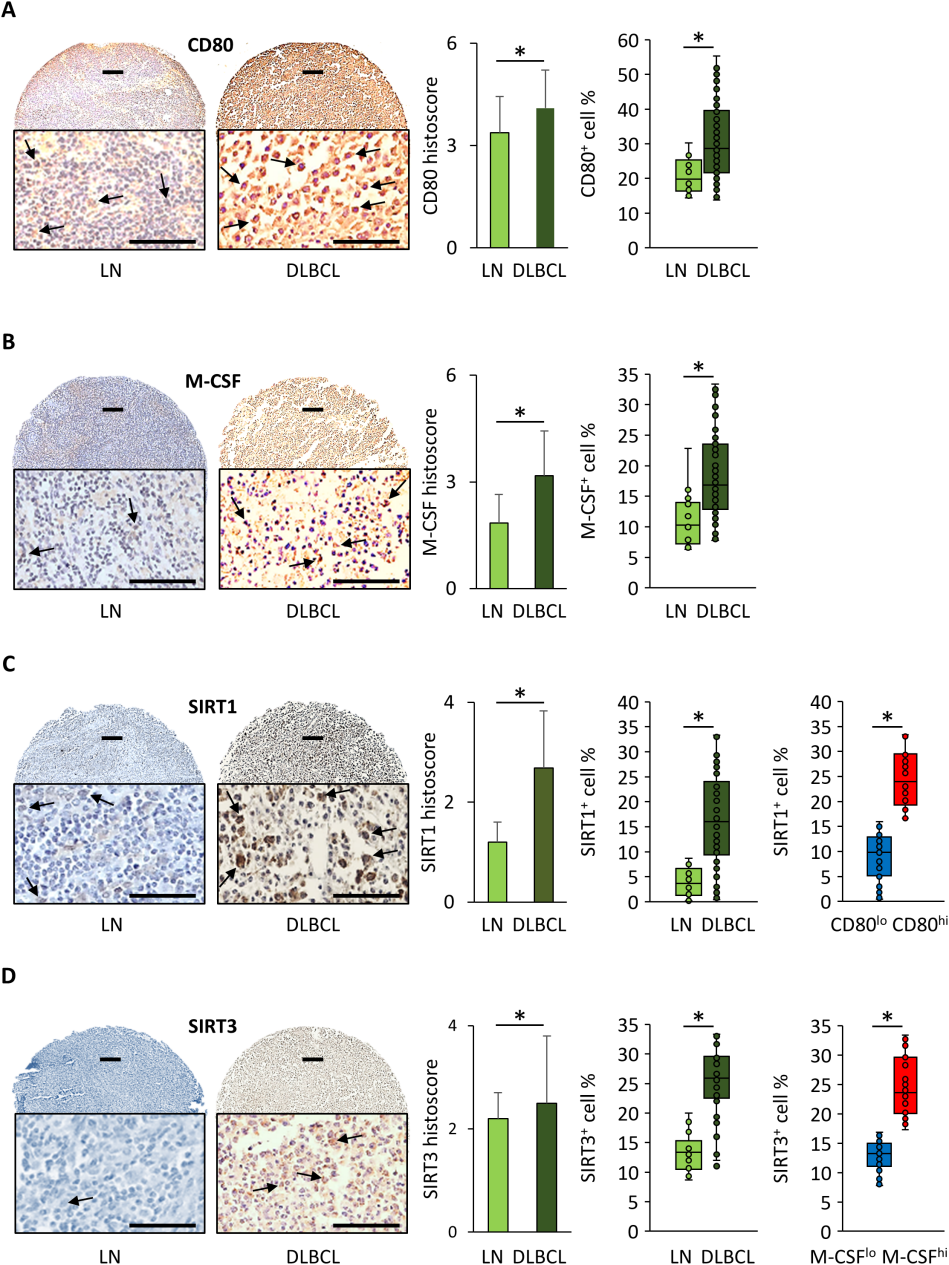
Immunohistochemical analysis of SIRT1, SIRT3, CD80 and M-CSF expression in DLBCL tumors and in non-tumor control tissue samples. **(A)** Representative images showing CD80 expression in normal lymph nodes (LN) and DLBCL samples. Quantitative analysis of the CD80 histoscore (left panel) and percentage of CD80^+^ cells (right panel) in DLBCL and LN samples. **(B)** Representative images showing M-CSF expression in normal lymph nodes (LN) and DLBCL samples. Quantitative analysis of the M-CSF histoscore (left panel) and percentage of M-CSF^+^ cells (right panel) in DLBCL and LN samples. **(C)** Representative images showing SIRT1 expression in normal lymph nodes (LN) and DLBCL samples. Quantitative analysis of the SIRT1 histoscore (left panel) and percentage of SIRT1^+^ cells (middle panel) in DLBCL and LN samples and percentage of SIRT1^+^ cells in the CD80^low^ and CD80^high^ DLBCL groups (right panel). **(D)** Representative images showing SIRT3 expression in normal lymph nodes (LN) and DLBCL samples. Quantitative analysis of the SIRT3 histoscore (left panel) and percentage of SIRT3^+^ cells (middle panel) in DLBCL and LN samples and percentage of SIRT3^+^ cells in the M-CSF^low^ and M-CSF^high^ DLBCL groups (right panel). Scale bars, 100 µm. *P ≤0.05 was considered significant.

The mean histoscore for CD80 (macrophage marker) was significantly higher in DLBCL samples than in normal lymph nodes (4.1 ± 1.1 vs 3.4 ± 1.1, p = 4.6e-02) as well as the mean percentage of CD80^+^ cells (30.6% ± 5.2 vs 20.8% ± 10.7, p = 7.3e-06) (**Figure 5A**).

Similarly, the mean histoscore for M-CSF (M2 macrophage marker) (3.2 ± 1.3 vs 1.8 ± 0.8, p = 7.2e-05) and the mean percentage of M-CSF^+^ cells (18.7% ± 7.3 vs 10.2% ± 3.3, p = 2.8e-05) were significantly higher in DLBCL samples than in normal lymph nodes (**Figure 5B**).

The mean SIRT1 histoscore and mean percentage of SIRT1^+^ cells were significantly higher in DLBCL than in lymph node samples (3.3 ± 1.3 vs 1.3 ± 0.5, p = 5.5e-19; and 16.4% ± 1.1 vs 2.8% ± 0.7, p = 1.5e-15, respectively) (**Figure 5C**). Then, to assess the correlation between CD80 and SIRT1 expression we classified the DLBCL samples into two groups using as threshold the median percentage of SIRT1^+^ cells (i.e. 16%), SIRT1^low^ (≤16%) and SIRT1^high^ (>16%), and the median percentage of CD80^+^ cells (i.e. 28.6%), CD80^low^ (≤28.6%) and CD80^high^ (>28.6%). We found that the percentage of SIRT1^+^ cells in the DLBCL microenvironment was higher in CD80^high^ than CD80^low^ samples (24.2% ± 5.4 vs 8.8% ± 4.9, p = 8.0e-20) (**Figure 5C**). This confirmed the association between SIRT1 and CD80 expression at the protein level.

The mean SIRT3 histoscore and mean percentage of SIRT3^+^ cells were significantly higher in DLBCL than in lymph node samples (2.5 ± 0.6 vs 2.2 ± 0.8, p = 1.6e-01; and 25.8% ± 5.3 vs 13.4% ± 3.6, p = 7.5e-12, respectively) (**Figure 5D**). Then, to assess the correlation between M-CSF and SIRT3 expression, we classified the samples into two groups using as threshold the median percentage of SIRT3^+^ cells (i.e. 25.9%) as threshold, SIRT3^low^ (≤25.9%) and SIRT3^high^ (>25.9%), and the median percentage of M-CSF^+^ cells (18.7%), M-CSF^low^ (≤18.7%) and M-CSF^high^ (>18.7%). We observed that the percentage of SIRT3^+^ cells in the DLBCL microenvironment was higher in the M-CSF^high^ than in the M-CSF^low^ group (29.7% ± 2.4 vs 21.6% ± 4.1, p = 2.6e-13) (**Figure 5D**). This finding supports the association between SIRT3 and M-CSF expression at the protein level.

Lastly, we evaluated the SIRT1/SIRT3 and CD80/MCSF ratios using the histoscore, which takes into account the percentage of positive cells and the expression intensity, to identify the most expressed metabolic factor and the most infiltrated macrophage subpopulation in DLBCL. We found that in the DLBCL microenvironment, there were more SIRT1^+^ than SIRT3^+^ cells and more CD80^+^ cells than M-CSF^+^ cells (ratios: 1.32 and 1.29, respectively; p ≤0.05).

Altogether, the IHC results obtained in an independent DLBCL cohort validated our bioinformatic results: increased expression of the SIRT1 and SIRT3 metabolic factors, higher infiltration of CD80^+^ than M-CSF^+^ cells, and correlation between SIRT1 and CD80 expression levels. On the basis of our findings, we hypothesize that in DLBCL, SIRT1 and SIRT3 might be important metabolic factors associated with different immune signatures.

### SIRT1 expression is correlated with those of CD80 and beclin-1, while SIRT3 expression is only correlated with that of M-CSF in the DLBCL macrophage microenvironment

**Figure 6:**
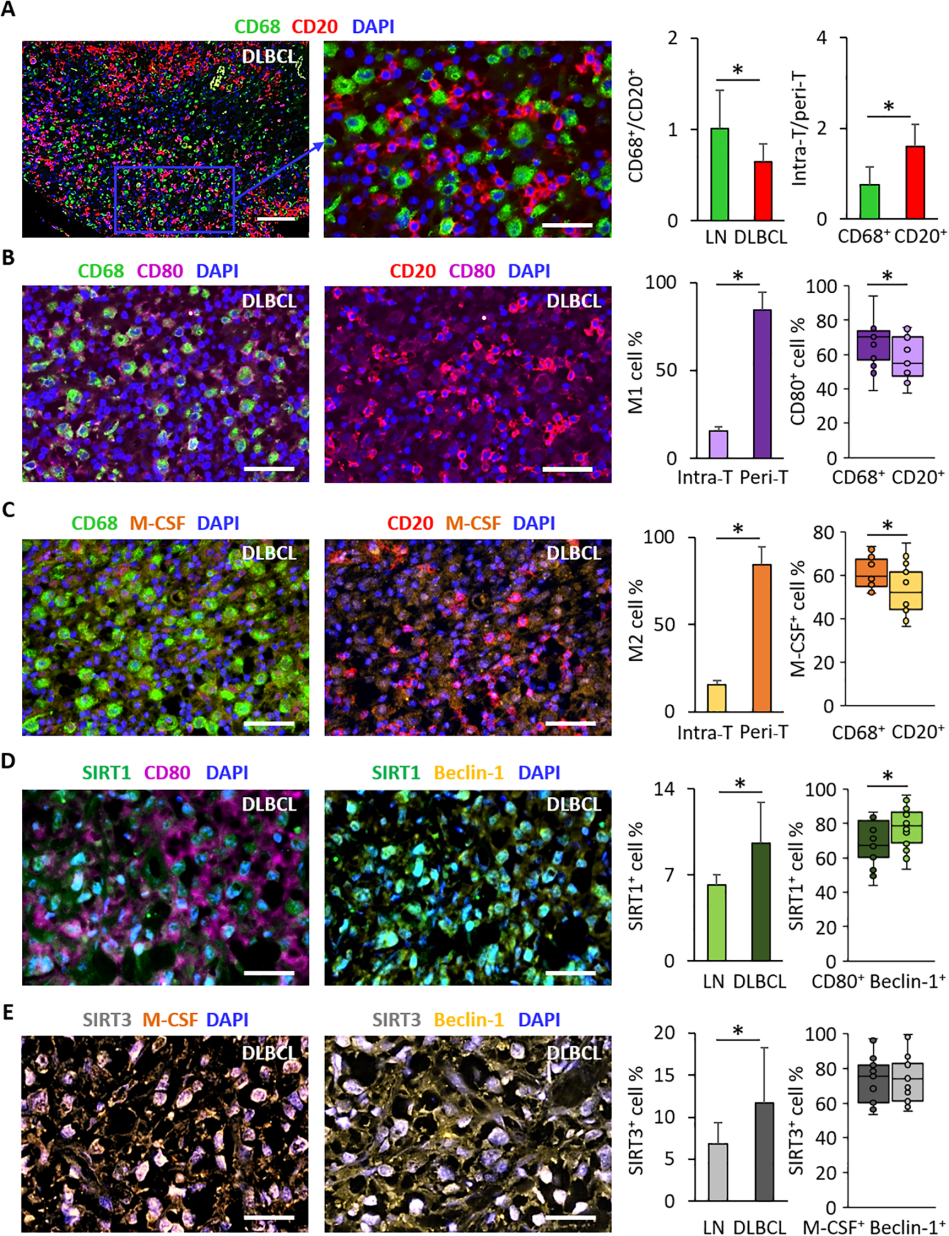
Immunofluorescence analysis of CD68 and CD20 distribution in DLBCL samples and of SIRT1, SIRT3, CD80, M-CSF and beclin-1 expression in the DLBCL macrophage microenvironment. **(A)** Representative images showing CD68 and CD20 expression and distribution in DAPI^+^ cells in DLBCL samples. Quantitative analysis of the CD68^+^/CD20^+^ ratio (left panel) and of the Intra-tumor (Intra-T)/Peripheral-tumor (Peri-T) ratio (right panel) of CD68^+^ and CD20^+^ cells in DLBCL samples. Representative images showing CD68 and CD20 expression and distribution in DAPI^+^ cells in DLBCL samples. **(B)** Representative images showing CD80 expression in CD68^+^ and CD20^+^ cells in DLBCL samples. Quantitative analysis of M1 macrophage (CD68^+^CD80^+^) distribution in the intra-tumor (intra-T) and peri-tumor (peri-T) areas (left panel) of DLBCL samples and of CD80^+^ cell percentage (right panel) in CD68^+^ and CD20^+^ cells in DLBCL samples. **(C)** Representative images showing M-CSF expression in CD68^+^ and CD20^+^ cells in DLBCL samples. Quantitative analysis of M2 macrophage (CD68^+^M-CSF^+^) distribution in the intra-tumor (intra-T) and peri-tumor (peri-T) areas (left panel) of DLBCL samples and of M-CSF^+^ cell percentage (right panel) in CD68^+^ and CD20^+^ cells in DLBCL samples. **(D)** Representative images showing SIRT1 expression in CD80^+^ and beclin-1^+^ cells in the DLBCL macrophage microenvironment. Quantitative analysis of SIRT1^+^ cell percentage in lymph node controls (LN) and in DLBCL (left panel) and of SIRT1^+^ cell percentage in CD80^+^ and beclin-1^+^ cells (right panel) in the DLBCL macrophage microenvironment. **(E)** Representative images showing SIRT3 expression in M-CSF^+^ and beclin-1^+^ cells in the DLBCL macrophage microenvironment. Quantitative analysis of SIRT3^+^ cell percentage in lymph node controls (LN) and in DLBCL (left panel) and of SIRT3^+^ cell percentage in M-CSF^+^ and beclin-1^+^ cells (right panel) in the DLBCL macrophage microenvironment. Scale bars, 100 µm. *P ≤0.05 was considered significant.

To investigate the co-localization and correlation of key tumor B cell and macrophage markers, we performed IF staining using the same TMA cohort. First, we determined the proportion of B cells (with CD20, a B cell surface marker and a malignant B cell marker) and macrophages (with CD68, a pan-macrophage marker) in DLBCL and control (LN) samples. The CD68^+^/CD20^+^ cell ratio was significantly lower in DLBCL than in control samples (0.6 ± 0.2 vs 1.0 ± 0.4, p = 0.006) (**Figure 6A**). Then, we explored the distribution of tumor B cells (CD20^+^) and macrophages (CD68^+^) in the DLBCL samples. We quantified these cells in the intra- and peri- tumor (intra-T and peri-T) areas and evaluated the intra-T/peri-T ratio. We found that the intra-T/peri-T ratio was significantly higher in CD20^+^ cells than in CD68^+^ cells (1.6 ± 0.5 vs 0.8 ± 0.4, p = 2.5e-06) (**Figure 6A**). These results suggest that in DLBCL samples, there are significantly more tumor B cells than infiltrating macrophages, with an inverted distribution of these cells in the intra-tumor and peri-tumor localization.

Due to the limited number of antibodies that could be used in combination for IF, we wanted to confirm that we could precisely differentiate M1 and M2 macrophages. To this aim, in addition to CD68 (pan-macrophage marker), CD80 (M1 marker) and M-CSF (M2 marker), we used an additional marker for each macrophage subtype (CD86 for M1 and CD206 for M2 macrophages). Then, we assessed the percentage of CD86^+^ and CD206^+^ cells in the CD68^+^CD80^+^ (M1) and CD68^+^M-CSF^+^ (M2) populations, respectively. As expected, we found that in DLBCL samples, 88% of CD68^+^CD80^+^ cells expressed CD86 and that 88.5% of CD68^+^M-CSF^+^ cells expressed CD206. In addition, only the percentage of CD68^+^CD80^+^ cells that expressed CD86, but not that of CD68^+^M-CSF^+^ cells that expressed CD206 was higher in DLBCL samples than control samples (**Supplementary Figures S6A, B**). These results suggest that we do not need to use both CD80 and CD86 and both M-CSF and CD206 to characterize M1 and M2 macrophages, respectively.

Second, we assessed in DLBCL samples the distribution/topography of M1 macrophages. Most M1 macrophages were localized in the peri-T area (84.2% ± 10.1) compared with the intra-T area (16.5% ± 2.4), p = 3.2e-10. Furthermore, as CD80 was overexpressed in the DLBCL samples compared with non-tumor controls (transcriptomic and IHC data), we wanted to know whether this pro-inflammatory marker was mostly expressed by tumor B cells (CD20^+^) or by tumor-infiltrating macrophages (CD68^+^). We found that more macrophages than tumor cells expressed CD80 (65.9% ± 12.9 vs 57.5% ± 12.1, p = 2.9e-02) (**Figure 6B**).

Third, we evaluated in DLBCL samples the distribution/topography of M2 macrophages. M2 macrophages were mostly localized in the peri-T area (83.3% ± 11.6) than intra-T area (16.7% ± 2.5), p = 8.7e-11. As M-CSF was overexpressed in DLBCL samples (although to a lower extent than CD80) compared with non-tumor controls, we determined whether it was expressed mainly by tumor B cells (CD20^+^) or by tumor-infiltrating macrophages (CD68^+^). M-CSF was overexpressed more by macrophages than by tumor B cells (61.2% ± 7.1 vs 53.1% ± 10.8, p = 4.1e-03) (**Figure 6C**). Moreover, in line with our IHC results and the study by Serna et al., we found a higher infiltration of CD80^+^ cells than M-CSF^+^ cells, suggesting a high inflammatory status in the DLBCL microenvironment.

Fourth, we examined the SIRT1^+^ cell percentage in DLBCL and control samples. In line with our bioinformatics and IHC results, SIRT1^+^ expression was higher in DLBCL than control samples (9.6 ± 3.3 vs 6.2 ± 0.8, p = 0.004). We obtained different percentages of SIRT1^+^ cells in DLBCL samples by IHC and IF (16.4% ± 1.1 and 9.6% ± 3.3). This could be explained by the use of adjacent TMA slides and differences in the IHC and IF protocols. However, both methods showed that the percentage of SIRT^+^ cells was higher in tumors than controls.

Then, we assessed the correlation between SIRT1 and CD80 (M1 macrophage marker) and beclin-1 (autophagy marker) in macrophages in the DLBCL microenvironment. Due to technical issues and antibody incompatibility and/or unavailability, we exploited the cell localization (intra-T and peri-T) and morphological characteristics. We quantified SIRT1 and CD80 expression in macrophage-rich areas (peri-T areas) identified by a pathologist. SIRT1^+^CD80^+^ cells were more abundant in DLBCL than in non-tumor controls (67.3% ± 12.8 vs 54.6% ± 14.9, p = 0.01) (data not shown), but not SIRT1^+^beclin-1^+^ cells (78.6% ± 11.7 vs 70.5% ± 16.5, p = 0.12) (data not shown). However, when we quantified their expression only in DLBCL samples, we found more SIRT1^+^beclin-1^+^ cells than SIRT1^+^CD80^+^ cells (76.9% ± 11.7 vs 54.6% ± 14.9, p = 0.01) (**Figure 6D**). This suggests that in the DLBCL macrophage microenvironment, there are more inflammatory cells with high SIRT1 metabolism related to the autophagy process.

Fifth, SIRT3^+^ cell percentage was higher in DLBCL than LN samples (11.7% ± 6.5 vs 6.8% ± 2.5, p = 0.034). In addition, to assess the correlations between SIRT3 and M-CSF (M2 macrophage marker) and beclin-1 (autophagy marker) in the DLBCL microenvironment, we analyzed SIRT3 and M-CSF expression in macrophage-rich peri-T areas, using cell localization and morphology (results validated by a pathologist). The percentage of SIRT3^+^M-CSF^+^ cells was higher in DLBCL than LN samples (75.5% ± 17.6 vs 62.2% ± 15.6, p = 0.046) (data not shown), but not the percentage of SIRT3^+^beclin-1^+^ cells (73.9% ± 12.8 vs 78.6% ± 18.0, p = 0.234) (data not shown). Moreover, in DLBCL samples, the percentages of SIRT3^+^beclin-1^+^ and SIRT3^+^M-CSF^+^ cells were similar (73.9% ± 12.8 vs 75.5% ± 17.6, p = 0.380) (**Figure 6F**). This suggests that in DLBCL macrophages, SIRT3 overexpression is correlated with an immunosuppressive profile, but not to autophagy.

Altogether, these IF results obtained on the same TMAs used for IHC, validated and complemented our bioinformatics and IHC results. In DLBCL, infiltration by CD80^+^ cells was higher than that by M-CSF^+^ cells, and correlated with the expression of SIRT1 and SIRT3 respectively. However, only SIRT1 expression correlated with a high autophagy profile.

### M1 and M2 macrophages crosstalk with tumor B cells and T cells in the DLBCL microenvironment

**Figure 7:**
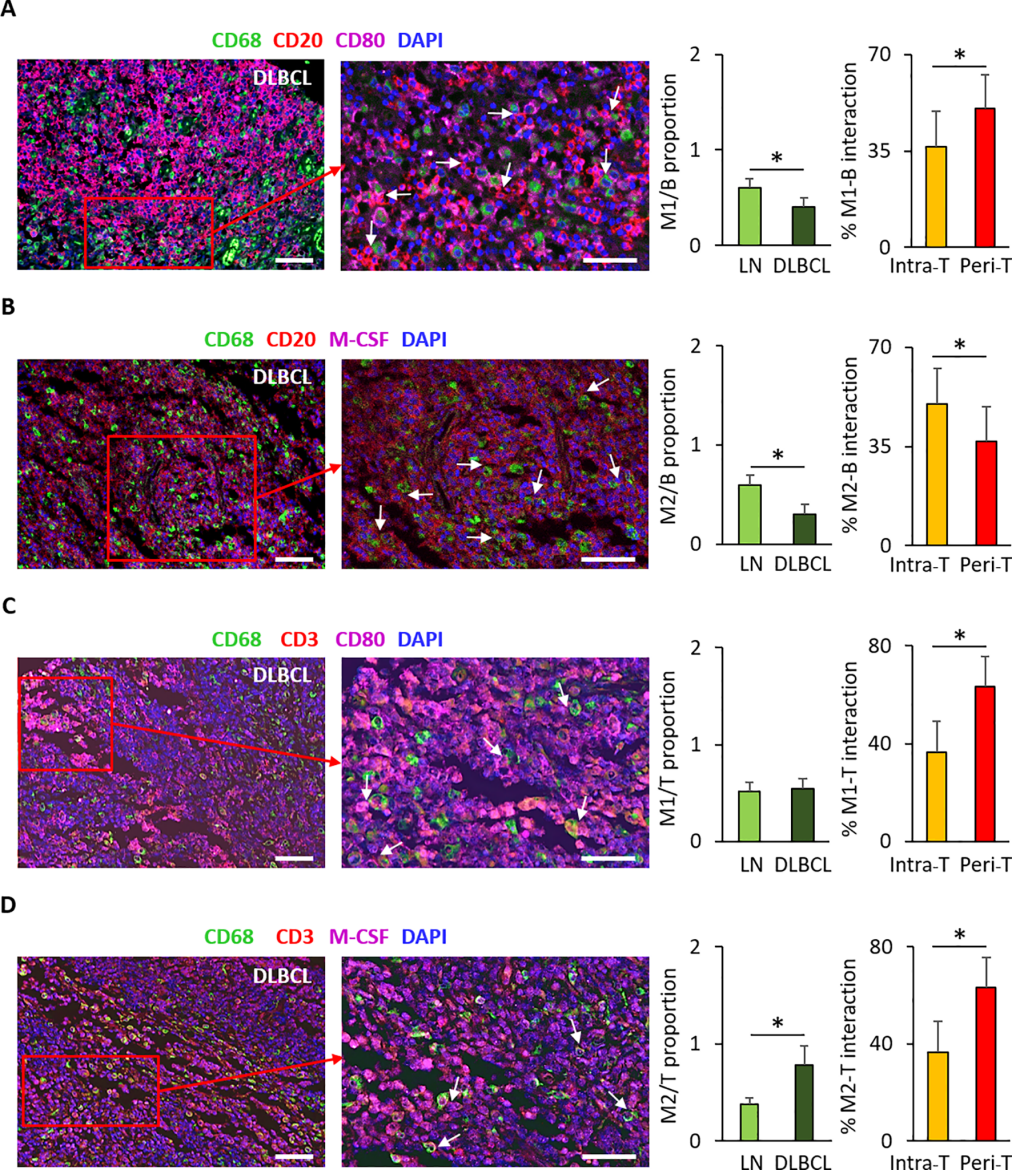
Immunofluorescence analysis of the proportion of M1 and M2 macrophage interactions with B cells and T cells in DLBCL and controls and their localization in the DLBCL microenvironment. **(A)** Representative images showing M1 macrophages (CD68^+^CD80^+^) adjacent to B cells (CD20^+^) in DLBCL samples. Quantitative analysis of the M1/B cell (CD68^+^CD80^+^/CD20^+^) ratio (left histogram) and of the percentage of adjacent M1 macrophages and B cells in the intra-tumor (intra-T) and peripheral-tumor (peri-T) areas (right histogram) in DLBCL samples. **(B)** Representative images showing M2 macrophages (CD68^+^M-CSF^+^) adjacent to B cells (CD20^+^) in DLBCL samples. Quantitative analysis of the M2/B cell (CD68^+^M-CSF^+^/CD20^+^) ratio (left histogram) and of the percentage of adjacent M2 macrophages and B cells in the intra-tumor (intra-T) and peripheral-tumor (peri-T) areas (right histogram) in DLBCL samples. **(C)** Representative images showing M1 (CD68^+^CD80^+^) macrophages adjacent to T cells (CD3^+^) in DLBCL samples. Quantitative analysis of the M1/T cell (CD68^+^CD80^+^/CD3^+^) ratio (left histogram) and of the percentage of adjacent M1 macrophages and T cells in the intra-tumor (intra-T) and peripheral-tumor (peri-T) areas (right histogram) in DLBCL samples. **(D)** Representative images showing M2 (CD68^+^M-CSF^+^) macrophages adjacent to T cells (CD3^+^) in DLBCL samples. Quantitative analysis of the M2/T cell (CD68^+^M-CSF^+^/CD3^+^) ratio (left histogram) and of the percentage of adjacent M2 macrophages and T cells in the intra-tumor (intra-T) and peripheral-tumor (peri-T) areas (right histogram) in DLBCL samples. Scale bars, 100 µm. *P ≤0.05.

To investigate the interaction of M1 and M2 macrophages with tumor B cells and T cells, we performed IF staining using the same TMA cohort. First, we determined the M1/B cell ratio using antibodies against CD68 and CD80 (M1 macrophages) and CD20 (B cell marker) in DLBCL and control (LN) samples. The M1/B cell ratio was significantly lower in DLBCL than control samples (0.4 ± 0.1 vs 0.6 ± 0.1, p = 0.01) (**Figure 7A**). Then, we estimated the percentage of tumor B cells (CD20^+^) that were adjacent to M1 macrophages (CD68^+^CD80^+^) in the intra-T and peri-T areas of the DLBCL samples. This percentage was significantly higher in the peri-T than intra-T area (50.4 ± 12.2 vs 36.6 ± 12.7, p = 0.05) (**Figure 7A**).

Similarly, we determined the M2/B cell ratio using antibodies against CD68 (pan-macrophage marker) and M-CSF (M2 macrophage marker) and CD20 (B cells marker) in DLBCL and control (LN) samples. As observed for the M1/B cell ratio, the M2/B cell ratio was significantly lower in DLBCL than control samples (0.3 ± 0.1 vs 0.6 ± 0.2, p = 0.01) (**Figure 7B**). Conversely, the percentage of B cells that were adjacent to M2 macrophages was significantly higher in the intra-T (where malignant B cells are more abundant) than peri-T area (50.0 ± 1.0 vs 36.8 ± 2.8, p = 3e-06) (**Figure 7B**).

Then, we evaluated the M1/T cell ratio using antibodies against CD68 and CD80 (M1 macrophages) and CD3 (T cell marker) in DLBCL and control (LN) samples. This ratio was similar in DLBCL and control samples (0.54 ± 0.2 vs 0.51 ± 0.1, p = 0.31) (**Figure 7C**). Conversely, the percentage of T cells (CD3^+^) adjacent to M1 macrophages (CD68^+^CD80^+^) was significantly higher in the peri-T than intra-T area of DLBCL (63.4 ± 3.7 vs 36.6 ± 3.7, p = 1e-19) (**Figure 7C**). These results suggest that in DLBCL samples, there is a significantly greater crosstalk between M1 macrophages and T cells in the peri-tumor area.

Lastly, we determined the M2/T cell ratio using antibodies against CD68 and M-CSF (M2 macrophages) and CD3 (T lymphocytes) and found that it was significantly higher in DLBCL than control samples (0.8 ± 0.2 vs 0.4 ± 0.1, p = 0.001) (**Figure 7D**). Similarly, the percentage of T cells adjacent to M2 macrophages was higher in the peri-T than intra-T area (63.6 ± 8.8 vs 36.4 ± 8.8, p = 5e-13) (**Figure 7D**). These results suggest that in DLBCL samples, there are significantly more interactions between M2 macrophages and T cell in the peri-tumor area.

Altogether, these results indicate the M1 macrophages interact with tumor B cells and T cells mainly in the peri-T area. Conversely, M2 macrophages interact with malignant B cells mainly in the intra-T area and with T cells mainly in the peri-T area where these cells are more abundant.

## Discussion

The innate immune microenvironment is the first line of immune defense. It also strongly influences the tumor landscape and prognosis, especially in hematological malignancies, such as DLBCL (38, 48–50). In DLBCL, changes in the innate immune cell composition (macrophages) and response (inflammation) require a substantial amount of energy. This would entail significant modifications in the expression of key metabolic factors (e.g. sirtuins, involved in many key physiological processes). Autophagy could also be exploited as a source of energy for ongoing fundamental cellular processes (51). High sirtuin expression is associated with poor prognosis in hematologic malignancies. However, the link between sirtuins and autophagy and their association with inflammation have been little documented. Only few studies have reported a link between inflammation-inhibiting/resolving functions in metabolic disorders, but not in cancer (15, 16, 52).

Here, we investigated the expression levels of SIRT1 and SIRT3 and of autophagy components in DLBCL samples, and their possible link with pro- or anti-inflammatory signatures. To do this, we analyzed already available datasets and validated our bioinformatics findings on a substantial independent cohort of DLBCL samples by IHC and IF.

Compared to non-tumor controls, DLBCL samples showed significantly higher *SIRT1*, *SIRT3* and *SIRT6* expression in their microenvironment. However, only *SIRT1* and *SIRT3* expression levels were correlated. We also observed a strong autophagy signature in the DLBCL microenvironment. Moreover, *SIRT1* and *SIRT3* were correlated with *CD80* and *MCSF* expression levels (M1 and M2 macrophage markers, respectively), and with M1 and M2 macrophage infiltration levels. Most autophagy components were positively correlated with *SIRT1* and *SIRT3* expression levels, which in turn, were correlated with the expression of distinct pro- and anti-inflammatory signatures, respectively. Together, these findings suggest that SIRT1/SIRT3 expression, autophagy activation and macrophage polarization converge to shape the inflammatory balance in the DLBCL microenvironment, thereby influencing tumor–immune cell interactions. This integrated axis highlights how metabolic and stress-response pathways coordinate with immune plasticity to sustain lymphoma progression. This clear SIRT1 and SIRT3 dichotomy might not prevent the crosstalk between SIRT1^+^M1 macrophages and SIRT3^+^M2 macrophages to shape the tumor microenvironment. Indeed, SIRT1^+^ M1 and SIRT3^+^ M2 macrophages with complementary metabolic programs and reciprocal cytokine signaling (TNF-α and IL-6 vs IL-10, TGF-β, respectively) might coexist in the DLBCL microenvironment. For instance, in a DLBCL cell–derived CCL5-conditioned niche, SIRT1^+^ M1 macrophages could deliver short bursts of inflammatory signals, while remaining metabolically adaptable, contributing to a localized immune response without progressing to chronic inflammation. Moreover, CCL5 promotes M2-like polarization, leading to the formation of a metabolite-rich, immunosuppressive niche that supports tumor survival (53, 54).

Lastly, *MTORC1* (known to play an essential role in the management of metabolic stress and energy balance in mammals) was overexpressed and enriched in our DLBCL cohort. It was also correlated with *SIRT1* and *BECN1* expression and with M1 macrophage infiltration.

These strong autophagy and metabolic signatures (including SIRT1 and SIRT3), mostly detected in the macrophage compartment, were not associated with poor survival of patients with DLBCL, unlike in previous studies (15, 18, 19, 55). This is likely due to the small publicly available cohort (n= 48) used for the transcriptomic analysis, which may have prevented us from achieving statistical significance.

Then, we validated most of our transcriptomic results in an independent cohort of patients with DLBCL and controls. We also investigated the topography (numeration and distribution) of tumor B cells (CD20^+^) and macrophages (CD68^+^). We found more tumor cells than macrophages in DLBCL, with a significantly higher abundance of macrophages in the peri-tumor areas (i.e. macrophage microenvironment). Next, by assessing the expression of CD80 and M-CSF in CD68^+^ and CD20^+^ cells in the macrophage microenvironment, we found significantly higher CD80 expression (pro-inflammation macrophage marker) in macrophages than in tumor B cells. Moreover, we examined the co-localization of SIRT1 and SIRT3 with CD80 and M-CSF (pro- and anti-inflammatory markers), respectively, and with beclin-1 (autophagy marker). Our results showed a significant difference in the expression of SIRT1 and SIRT3 in DLBCL compared with non-tumor controls, and in their correlation with CD80 and M-CSF expression, respectively. Moreover, in DLBCL samples, SIRT1, but not SIRT3, was significantly correlated with beclin-1 expression. Lastly, when we examined the qualitative (localization) and quantitative (percentage and proportion) interaction of M1 and M2 macrophages with tumor B cells (CD20^+^) and T cells (CD3^+^), we found that M1 macrophages were in contact with malignant B cells mainly in the peri-tumor area. Conversely, M2 macrophage interaction localization changed in function of the area where cells were more abundant: intra-tumoral with tumor B cells and peri-tumoral with T cells.

Altogether, this study is the first to demonstrate a direct link between sirtuin expression, macrophage subtype polarization, and autophagy in the DLBCL immune microenvironment. We show an increased metabolic activity related to pro-inflammatory components associated with SIRT1 (more specifically associated with autophagy) and to anti-inflammatory components associated with SIRT3. This extends sirtuin role in DLBCL from metabolic factor to immune regulation.

In physiological conditions, autophagy is used by macrophages to regulate immune signaling pathways, as a way to limit inflammation (56). In pathological conditions, some interesting studies are in line with our results. Sadria et al., developed a computational model that simulates the interactions among key regulators, such as mTOR, and predicts their dynamics. They found a clinically important role of mTORC1 upregulation and its tight link with SIRT1-activating compounds and premature autophagy despite nutrient abundance in age-related diseases (e.g. cancer) (57). Indeed, in up to 70% of all human tumors, malignant cells adapt to a deregulation of the balance between anabolic (energy-consuming) and catabolic (energy-producing) processes through hyperactivation of mTORC1 signaling by most pro-oncogenic signaling pathways to promote tumor growth and autophagy regulation (58–60). In a mouse model of atherosclerosis, a chronic heart inflammatory disease, treatment with araloside C (a natural anti-inflammatory component) activates and upregulates SIRT1 signaling that then mediates macrophage autophagy and polarization toward the anti-inflammatory M2 macrophage type (61). In DLBCL (characterized by chronic inflammation), our results suggest that autophagy in inflammatory macrophages is specifically correlated with SIRT1 overexpression (and not with SIRT3) and with mTOR signaling pathway enrichment. As macrophages are the most abundant tumor-infiltrating immune cell population in DLBCL (38), tumor cells might specifically upregulate SIRT1 (a major regulator of energy metabolism and of inflammation) in M1 macrophages to enhance their autophagy activity and inhibit the inflammatory response, thus promoting cancer development (8, 9, 36, 52, 62). This upregulation may reflect the tumor cell adaptation to immune pressure and associated microenvironmental stressors, such as hypoxia and oxidative stress, conditions known to drive SIRT1 activation in aggressive tumors. Tumor cells can hijack immune functions by conditioning tumor-associated macrophages through CSF-1 expression and by exploiting hypoxia- and lactate-driven cytokine secretion to establish an immunosuppressive milieu. This environment blunts the immune surveillance and provides metabolic by-products (e.g., lactate, amino acids, lipids) that tumor cells can repurpose for energy and growth. In parallel, tumor-associated macrophages increase their metabolic activity by consuming glutamine and fatty acids, while reshaping the tumor microenvironment by depleting amino acids needed by effector T cells, secreting lactate, and releasing cytokines (IL-6, TNF, CCL5, CCL18). These metabolites and signals enrich the tumor microenvironment with substrates that tumor cells can scavenge and metabolize (63). Moreover, the tumor cell metabolic rewiring involves SIRT3 (mitochondrial) and SIRT6 (nuclear), which connect bioenergetic regulation with glycolysis and lipid metabolism, supporting survival signaling. This may address the high proliferative and metabolic demands of DLBCL. By integrating metabolic and stress-response signals, SIRT1 may facilitate both immune evasion and sustained tumor growth. Mechanistically, SIRT1-mediated AMPK activation might counterbalance mTORC1 signaling, attenuating inflammation and favoring autophagy (64, 65). Concomitantly, the host immune system might use SIRT1 overexpression to integrate energy metabolism necessary for the pro-inflammatory activity of macrophages (the most abundant immune cells in DLBCL) to promote a cancer-limiting inflammatory response.

Epigenetic modifications/reprogramming and SIRT1 genetic alterations could also contribute to modulate macrophage functions. All these events would imply an adaptation at the epigenetic and genetic levels, as usually observed in lymphoma (2, 3).

**Figure 8:**
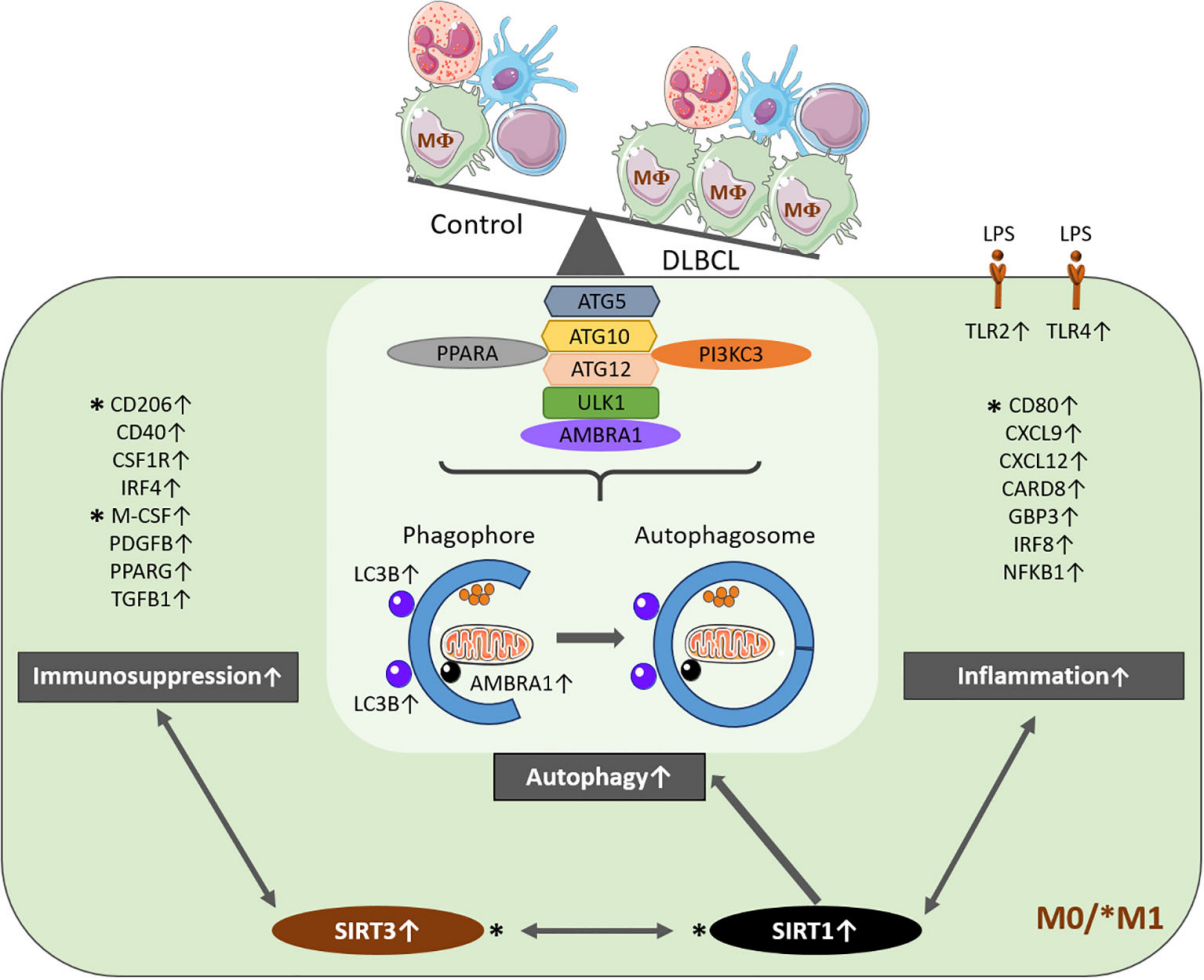
Schematic model of the molecular events underlying SIRT1- and SIRT3-related upregulation of autophagy in macrophages of the DLBCL microenvironment. In DLBCL, M0 and M1 macrophage infiltration is increased compared with non-tumor controls and with infiltration by other immune cells. Most of the key autophagy components are upregulated in tumor-infiltrating M0 and M1 macrophages. *SIRT1* and *SIRT3* (two metabolic sensors) are upregulated in these two macrophage subtypes in the DLBCL microenvironment. *SIRT1* expression is correlated with that of most of the autophagy components analyzed (*AMBRA1, ATG5, ATG10, ATG12, LC3B, PI3KC3, PPARA* and *ULK1*). Moreover, *SIRT1* expression is also correlated with that of several inflammation factors belonging to the inflammasome (*CARD8, GBP3, IRF8*, and *NFKB1*) and inflammation factors (*CD80, CXCL9, CXCL11, CXCL12, TLR2* and *TLR4*). *SIRT3* expression is correlated with several immunosuppressive factors (*CD206, CD40, CSF1R, MCSF, PDGFB, PPARA* and *TGFB1*). This suggests that SIRT1 and SIRT3 might influence the inflammatory state of the DLBCL microenvironment. Some key results were validated at the protein level (marked with *).

We conclude that macrophages in the DLBCL microenvironment may increase their metabolic activity in association with the upregulation of SIRT1 and SIRT3 (**Figure 8**). The observed correlation between *SIRT1* expression, *MTORC1* signaling, and M1 macrophage infiltration suggests a potential link to chronic inflammation in DLBCL, which may contribute to metabolic adaptations that support tumor survival. Our findings align with previous TCGA DLBCL profiling data (66). Previous studies showed that SIRT1 expression in tumor cells is a negative prognostic factor (15) and that SIRT3 promotes tumor growth in ATM-deficient DLBCL (13). Our study shifts the focus from tumor cells to the immune microenvironment, by providing new evidence that autophagy components and metabolic sensors, including SIRT1 and 3, are specifically upregulated in the DLBCL macrophage microenvironment and contribute to shape the inflammatory state of these crucial immune cells.

Although TMAs are considered a powerful way to investigate *in situ* protein expression (IHC) and interactions (IF), our study has several limitations. First, the relatively small publicly available cohort (n = 48 DLBCL samples) used for the transcriptomic analysis may have limited the statistical power, preventing the detection of robust survival associations. Second, the reliance on TMAs without fresh patient samples restricted our ability to validate findings at the single-cell level or to perform functional assays. Third, the absence of clinical treatment response data (e.g., first- or second-line therapy outcomes) for the TCGA cohort and the TMA samples prevented comparisons between responders and non-responders. Such data would have provided valuable insights into the prognostic and predictive relevance of SIRT1, SIRT3 and autophagy markers in DLBCL.

Future studies should focus on the therapeutic targeting and/or modulation of SIRT1 and its epigenetic modulators (such as NCAPD3) in macrophages of patients with DLBCL. Indeed, previous studies showed that these molecules play a crucial role in lymphoma (67, 68). Small−molecule SIRT1 modulators, such as SRT1720 and SRT2104 (currently advancing into the clinical translation phase), show promise in inducing lymphoma cell death and modulating metabolic and longevity pathways (11). Natural phytochemical, including resveratrol, fisetin, quercetin and curcumin, also activate SIRT1, improving metabolic regulation and insulin sensitivity while modulating autophagy, apoptosis, and ferroptosis (11, 68–70). SIRT1 modulation in lymphoma is doable; however, challenges remain, such as off−target effects and context−dependent immune consequences. The evolving clinical landscape of SIRT inhibitors (such as cambinol) underscores both their therapeutic potential (14, 71) and the need of careful translational evaluation. This approach might open new avenues for DLBCL treatment, while reducing toxicity (72–75). Additionally, the development of bioinformatics models that take into account tissue specificity and patient characteristics (e.g. sex, age, macrophage infiltration) could help to identify therapeutic strategies that target metabolic pathways linked to abnormal inflammation.

## Data Availability

The original contributions presented in the study are included in the article/**Supplementary Material**. Further inquiries can be directed to the corresponding author.

## Supplementary material

Supplementary Figure 1 The expression of most differentially regulated genes **(A)** and inflammation/autophagy-related genes **(B)** was analyzed using the raw transcriptomic data of DLBCL versus spleen samples published in Serna, L et al., 2023 (**Supplementary Table S2**) and those displayed in this current study (**Supplementary Table S3**). Volcano plots were generated with VulcaNoseR (76).

Supplementary Figure 2 Changes in each immune cell type proportion in 47 DLBCL samples (TCGA database) and 337 spleen tissue samples (GTEx database) assessed with the TIMER2.0 tool. **(A)** Immune infiltration estimation in arbitrary units (A.U.) of B cells **(B)**, CD4^+^ T cells, CD8^+^ T cells, macrophages (MФ), natural killer (NK) cells, and other cells using the EPIC algorithm. **(B)** Immune infiltration estimation (A.U.) of B cells **(B)**, CD8^+^ T cells, CD4^+^ T cells, regulatory T cells (Tregs), natural killer (NK) cells, monocytes (Mo), M0, M1 and M2 macrophages, mature dendritic cells (mDC), and neutrophils (Neuφ) using the CYBERSORT algorithm. **(C)** Proportion (assessed with the TIMER2.0 tool) of CD8^+^ T cells, CD4^+^ T cells, macrophages (MФ), neutrophils (Neuφ), mature dendritic cells (mDC), monocytes (Mo), natural killer cells (NK) and B cells **(B)** using the EPIC and CIBERSORT algorithms. One-way ANOVA was used for the comparison of the different immune cell types and proportions. P ≤0.05 was considered significant.

Supplementary Figure 3 Violin plots displaying the expression profiles of CD68 **(A)**, CD80 **(B)**, M-CSF **(C)**, SIRT1 **(D)** and SIRT3 **(E)** in DLBCL samples classified according to their stage.

Supplementary Figure 4 Schematic overview of the deconvolution-based analysis in GEPIA2021. **(A)** Bulk gene expression profiles from TCGA and GTEx datasets are deconvoluted using CIBERSORT, EPIC, or quanTIseq to estimate the cell-type proportion matrix and cell-type-specific expression profiling matrix across samples. **(B)** Proportion comparison: cell type proportions (e.g., CD4^+^ T cells, B cells, NK cells) are quantitatively compared across groups or conditions using the ANOVA test. **(C)** Cell type-level differential expression (DE): gene expression values (e.g., CD19) are inferred in individual cell types (e.g., B vs. NK cells), allowing differential expression testing in a specific cellular context. **(D)** Proportion correlation: pairwise correlations between cell type proportions (e.g., CD4^+^ T cells vs. B cells) across samples are visualized and analyzed using Pearson correlations. **(E)** Cell type-level survival analysis: samples are stratified based on the abundance of a given cell type (e.g., high vs. low NK cell infiltration), and survival outcomes are compared using the Kaplan–Meier analysis and the log-rank test.

Supplementary Figure 5 **(A)** Kaplan-Meier survival curve plots based on the expression status of the *AMBRA1, ATG5, ATG10, ATG12, LC3B, PIK3C3, PPARA, SIRT1, SIRT3* and *ULK1* genes in DLBCL, evaluated in this study (**Supplementary Table S3**). **(B)**. Survival maps based on the comparison of the survival contribution of the *AMBRA1, ATG5, ATG10, ATG12, LC3B, PIK3C3, PPARA, SIRT1, SIRT3* and *ULK1* genes, estimated using the Mantel–Cox test.

Supplementary Figure 6 Phenotypic validation of M1 and M2 macrophage subtypes in DLBCL and controls. **(A)** Representative image showing CD68, CD86 and CD86 co-expression in DAPI^+^ cells in DLBCL samples. Quantitative analysis of CD68^+^CD86^+^CD80^+^ cells in lymph nodes (LN) and DLBCL samples. **(B)** Representative image showing CD68, CD206 and M-CSF co-expression in DAPI^+^ cells in DLBCL samples. Quantitative analysis of CD68^+^CD206^+^M-CSF^+^ cells in lymph nodes (LN) and DLBCL samples. Scale bars, 100 µm. * P ≤0.05 was considered significant.

Supplementary Table 1 List of the characteristics of patients with DLBCL from the TCGA database.

Supplementary Table 2 List of the characteristics of the patients whose tumor samples were included in the tissue microarrays used for the immunohistochemistry analysis.

Supplementary Table 3 Manually curated list of the differentially expressed autophagy-related genes in DLBCL samples and spleen samples with their respective fold change and p values. P ≤0.05 was considered significant.

## References

[1] Armitage JO Gascoyne RD Lunning MA Cavalli F . Non-Hodgkin lymphoma. Lancet. (2017) 390:298–310. doi: 10.1016/S0140-6736(16)32407-2, PMID: 28153383

[2] Amin R Braza MS . The follicular lymphoma epigenome regulates its microenvironment. J Exp Clin Cancer Res. (2022) 41:21. doi: 10.1186/s13046-021-02234-9, PMID: 35022084 PMC8753841

[3] Ochando J Braza MS . T follicular helper cells: a potential therapeutic target in follicular lymphoma. Oncotarget. (2017) 8:112116–31. doi: 10.18632/oncotarget.22788, PMID: 29340116 PMC5762384

[4] Basso K Dalla-Favera R . Germinal centres and B cell lymphomagenesis. Nat Rev Immunol. (2015) 15:172–84. doi: 10.1038/nri3814, PMID: 25712152

[5] de Leval L Harris NL . Variability in immunophenotype in diffuse large B-cell lymphoma and its clinical relevance. Histopathology. (2003) 43:509–28. doi: 10.1111/j.1365-2559.2003.01758.x, PMID: 14636252

[6] Intlekofer AM Younes A . Precision therapy for lymphoma--current state and future directions. Nat Rev. (2014) 11:585–96. doi: 10.1038/nrclinonc.2014.137, PMID: 25135367

[7] Miao Y Medeiros LJ Li Y Li J Young KH . Genetic alterations and their clinical implications in DLBCL. Nat Rev. (2019) 16:634–52. doi: 10.1038/s41571-019-0225-1, PMID: 31127191

[8] Chalkiadaki A Guarente L . The multifaceted functions of sirtuins in cancer. Nat Rev Cancer. (2015) 15:608–24. doi: 10.1038/nrc3985, PMID: 26383140

[9] Houtkooper RH Pirinen E Auwerx J . Sirtuins as regulators of metabolism and healthspan. Nat Rev Mol Cell Biol. (2012) 13:225–38. doi: 10.1038/nrm3293, PMID: 22395773 PMC4872805

[10] Wu QJ Zhang TN Chen HH Yu XF Lv JL Liu YY . The sirtuin family in health and disease. Signal transduction targeted Ther. (2022) 7:402. doi: 10.1038/s41392-022-01257-8, PMID: 36581622 PMC9797940

[11] Chang N Li J Lin S Zhang J Zeng W Ma G . Emerging roles of SIRT1 activator, SRT2104, in disease treatment. Sci Rep. (2024) 14:5521. doi: 10.1038/s41598-024-55923-8, PMID: 38448466 PMC10917792

[12] Nahimana A Attinger A Aubry D Greaney P Ireson C Thougaard AV . The NAD biosynthesis inhibitor APO866 has potent antitumor activity against hematologic Malignancies. Blood. (2009) 113:3276–86. doi: 10.1182/blood-2008-08-173369, PMID: 19196867

[13] Bhalla K Jaber S Reagan K Hamburg A Underwood KF Jhajharia A . SIRT3, a metabolic target linked to ataxia-telangiectasia mutated (ATM) gene deficiency in diffuse large B-cell lymphoma. Sci Rep. (2020) 10:21159. doi: 10.1038/s41598-020-78193-6, PMID: 33273545 PMC7712916

[14] Heltweg B Gatbonton T Schuler AD Posakony J Li H Goehle S . Antitumor activity of a small-molecule inhibitor of human silent information regulator 2 enzymes. Cancer Res. (2006) 66:4368–77. doi: 10.1158/0008-5472.CAN-05-3617, PMID: 16618762

[15] Jang KY Hwang SH Kwon KS Kim KR Choi HN Lee NR . SIRT1 expression is associated with poor prognosis of diffuse large B-cell lymphoma. Am J Surg Pathol. (2008) 32:1523–31. doi: 10.1097/PAS.0b013e31816b6478, PMID: 18724249

[16] Li M Chiang YL Lyssiotis CA Teater MR Hong JY Shen H . Non-oncogene addiction to SIRT3 plays a critical role in lymphomagenesis. Cancer Cell. (2019) 35:916–31.e9. doi: 10.1016/j.ccell.2019.05.002, PMID: 31185214 PMC7534582

[17] Yang J Li Y Zhang Y Fang X Chen N Zhou X . Sirt6 promotes tumorigenesis and drug resistance of diffuse large B-cell lymphoma by mediating PI3K/Akt signaling. J Exp Clin Cancer Res. (2020) 39:142. doi: 10.1186/s13046-020-01623-w, PMID: 32711549 PMC7382040

[18] Yu W Denu RA Krautkramer KA Grindle KM Yang DT Asimakopoulos F . Loss of SIRT3 provides growth advantage for B cell Malignancies. J Biol Chem. (2016) 291:3268–79. doi: 10.1074/jbc.M115.702076, PMID: 26631723 PMC4751373

[19] Huang FT Sun J Zhang L He X Zhu YH Dong HJ . Role of SIRT1 in hematologic Malignancies. J Zhejiang Univ Science. (2019) 20:391–8. doi: 10.1631/jzus.B1900148, PMID: 31090265 PMC6568226

[20] Dilmac S Kuscu N Caner A Yildirim S Yoldas B Farooqi AA . SIRT1/FOXO signaling pathway in breast cancer progression and metastasis. Int J Mol Sci. (2022) 23:10227. doi: 10.3390/ijms231810227, PMID: 36142156 PMC9499652

[21] Wang Z Yuan H Roth M Stark JM Bhatia R Chen WY . SIRT1 deacetylase promotes acquisition of genetic mutations for drug resistance in CML cells. Oncogene. (2013) 32:589–98. doi: 10.1038/onc.2012.83, PMID: 22410779 PMC3376246

[22] Jin Y Cao Q Chen C Du X Jin B Pan J . Tenovin-6-mediated inhibition of SIRT1/2 induces apoptosis in acute lymphoblastic leukemia (ALL) cells and eliminates ALL stem/progenitor cells. BMC cancer. (2015) 15:226. doi: 10.1186/s12885-015-1282-1, PMID: 25884180 PMC4394409

[23] Li L Ye S Yang M Yu W Fan Z Zhang H . SIRT1 downregulation enhances chemosensitivity and survival of adult T-cell leukemia-lymphoma cells by reducing DNA double-strand repair. Oncol Rep. (2015) 34:2935–42. doi: 10.3892/or.2015.4287, PMID: 26398583

[24] Bhalla S Gordon LI . Functional characterization of NAD dependent de-acetylases SIRT1 and SIRT2 in B-Cell Chronic Lymphocytic Leukemia (CLL). Cancer Biol Ther. (2016) 17:300–9. doi: 10.1080/15384047.2016.1139246, PMID: 26794150 PMC4847985

[25] Dal Bo M D’Agaro T Gobessi S Zucchetto A Dereani S Rossi D . The SIRT1/TP53 axis is activated upon B-cell receptor triggering via miR-132 up-regulation in chronic lymphocytic leukemia cells. Oncotarget. (2015) 6:19102–17. doi: 10.18632/oncotarget.3905, PMID: 26036258 PMC4662478

[26] Kim TS Jin YB Kim YS Kim S Kim JK Lee HM . SIRT3 promotes antimycobacterial defenses by coordinating mitochondrial and autophagic functions. Autophagy. (2019) 15:1356–75. doi: 10.1080/15548627.2019.1582743, PMID: 30774023 PMC6628940

[27] Jia L Dourmashkin RR Allen PD Gray AB Newland AC Kelsey SM . Inhibition of autophagy abrogates tumour necrosis factor alpha induced apoptosis in human T-lymphoblastic leukaemic cells. Br J haematol. (1997) 98:673–85. doi: 10.1046/j.1365-2141.1997.2623081.x, PMID: 9332326

[28] Mizushima N Levine B Cuervo AM Klionsky DJ . Autophagy fights disease through cellular self-digestion. Nature. (2008) 451:1069–75. doi: 10.1038/nature06639, PMID: 18305538 PMC2670399

[29] Rao S Tortola L Perlot T Wirnsberger G Novatchkova M Nitsch R . A dual role for autophagy in a murine model of lung cancer. Nat Commun. (2014) 5:3056. doi: 10.1038/ncomms4056, PMID: 24445999

[30] White E . Deconvoluting the context-dependent role for autophagy in cancer. Nat Rev Cancer. (2012) 12:401–10. doi: 10.1038/nrc3262, PMID: 22534666 PMC3664381

[31] Jia L Gopinathan G Sukumar JT Gribben JG . Blocking autophagy prevents bortezomib-induced NF-kappaB activation by reducing I-kappaBalpha degradation in lymphoma cells. PLoS One. (2012) 7:e32584. doi: 10.1371/journal.pone.0032584, PMID: 22393418 PMC3290566

[32] Mathew R Karp CM Beaudoin B Vuong N Chen G Chen HY . Autophagy suppresses tumorigenesis through elimination of p62. Cell. (2009) 137:1062–75. doi: 10.1016/j.cell.2009.03.048, PMID: 19524509 PMC2802318

[33] Wu WK Coffelt SB Cho CH Wang XJ Lee CW Chan FK . The autophagic paradox in cancer therapy. Oncogene. (2012) 31:939–53. doi: 10.1038/onc.2011.295, PMID: 21765470

[34] Degenhardt K Mathew R Beaudoin B Bray K Anderson D Chen G . Autophagy promotes tumor cell survival and restricts necrosis, inflammation, and tumorigenesis. Cancer Cell. (2006) 10:51–64. doi: 10.1016/j.ccr.2006.06.001, PMID: 16843265 PMC2857533

[35] Levine B Mizushima N Virgin HW . Autophagy in immunity and inflammation. Nature. (2011) 469:323–35. doi: 10.1038/nature09782, PMID: 21248839 PMC3131688

[36] Mills EL Kelly B O’Neill LAJ . Mitochondria are the powerhouses of immunity. Nat Immunol. (2017) 18:488–98. doi: 10.1038/ni.3704, PMID: 28418387

[37] Nakahira K Haspel JA Rathinam VA Lee SJ Dolinay T Lam HC . Autophagy proteins regulate innate immune responses by inhibiting the release of mitochondrial DNA mediated by the NALP3 inflammasome. Nat Immunol. (2011) 12:222–30. doi: 10.1038/ni.1980, PMID: 21151103 PMC3079381

[38] Serna L Azcoaga P Brahmachary M Caffarel MM Braza MS . Diffuse large B-cell lymphoma microenvironment displays a predominant macrophage infiltrate marked by a strong inflammatory signature. Front Immunol. (2023) 14:1048567. doi: 10.3389/fimmu.2023.1048567, PMID: 37205092 PMC10185825

[39] Tang Z Li C Kang B Gao G Li C Zhang Z . GEPIA: a web server for cancer and normal gene expression profiling and interactive analyses. Nucleic Acids Res. (2017) 45:W98–W102. doi: 10.1093/nar/gkx247, PMID: 28407145 PMC5570223

[40] Li C Tang Z Zhang W Ye Z Liu F . GEPIA2021: integrating multiple deconvolution-based analysis into GEPIA. Nucleic Acids Res. (2021) 49:W242–W6. doi: 10.1093/nar/gkab418, PMID: 34050758 PMC8262695

[41] Li T Fu J Zeng Z Cohen D Li J Chen Q . TIMER2.0 for analysis of tumor-infiltrating immune cells. Nucleic Acids Res. (2020) 48:W509–W14. doi: 10.1093/nar/gkaa407, PMID: 32442275 PMC7319575

[42] Sturm G Finotello F Petitprez F Zhang JD Baumbach J Fridman WH . Comprehensive evaluation of transcriptome-based cell-type quantification methods for immuno-oncology. Bioinf (Oxford England). (2019) 35:i436–i45. doi: 10.1093/bioinformatics/btz363, PMID: 31510660 PMC6612828

[43] Kotlov N Bagaev A Revuelta MV Phillip JM Cacciapuoti MT Antysheva Z . Clinical and biological subtypes of B-cell lymphoma revealed by microenvironmental signatures. Cancer discovery. (2021) 11:1468–89. doi: 10.1158/2159-8290.CD-20-0839, PMID: 33541860 PMC8178179

[44] Klionsky DJ Petroni G Amaravadi RK Baehrecke EH Ballabio A Boya P . Autophagy in major human diseases. EMBO J. (2021) 40:e108863. doi: 10.15252/embj.2021108863, PMID: 34459017 PMC8488577

[45] Jablonski KA Amici SA Webb LM Ruiz-Rosado Jde D Popovich PG Partida-Sanchez S . Novel markers to delineate murine M1 and M2 macrophages. PloS One. (2015) 10:e0145342. doi: 10.1371/journal.pone.0145342, PMID: 26699615 PMC4689374

[46] Tremble LF McCabe M Walker SP McCarthy S Tynan RF Beecher S . Differential association of CD68(+) and CD163(+) macrophages with macrophage enzymes, whole tumour gene expression and overall survival in advanced melanoma. Br J cancer. (2020) 123:1553–61. doi: 10.1038/s41416-020-01037-7, PMID: 32843682 PMC7653046

[47] Murray PJ . Macrophage polarization. Annu Rev Physiol. (2017) 79:541–66. doi: 10.1146/annurev-physiol-022516-034339, PMID: 27813830

[48] Caffarel MM Braza MS . Microglia and metastases to the central nervous system: victim, ravager, or something else? J Exp Clin Cancer Res. (2022) 41:327. doi: 10.1186/s13046-022-02535-7, PMID: 36411434 PMC9677912

[49] Cioroianu AI Stinga PI Sticlaru L Cioplea MD Nichita L Popp C . Tumor microenvironment in diffuse large B-cell lymphoma: role and prognosis. Analytical Cell Pathol (Amsterdam). (2019) 2019:8586354. doi: 10.1155/2019/8586354, PMID: 31934533 PMC6942707

[50] Zhou H Zheng C Huang DS . A prognostic gene model of immune cell infiltration in diffuse large B-cell lymphoma. PeerJ. (2020) 8:e9658. doi: 10.7717/peerj.9658, PMID: 32844062 PMC7414766

[51] Oh JE Lee HK . Pattern recognition receptors and autophagy. Front Immunol. (2014) 5:300. doi: 10.3389/fimmu.2014.00300, PMID: 25009542 PMC4070062

[52] Kauppinen A Suuronen T Ojala J Kaarniranta K Salminen A . Antagonistic crosstalk between NF-kappaB and SIRT1 in the regulation of inflammation and metabolic disorders. Cell signalling. (2013) 25:1939–48. doi: 10.1016/j.cellsig.2013.06.007, PMID: 23770291

[53] Liu J Geng X Hou J Wu G . New insights into M1/M2 macrophages: key modulators in cancer progression. Cancer Cell Int. (2021) 21:389. doi: 10.1186/s12935-021-02089-2, PMID: 34289846 PMC8296555

[54] Manfroi B De Grandis M Moreaux J Tabruyn S Mayol JF Quintero M . The microenvironment of DLBCL is characterized by noncanonical macrophages recruited by tumor-derived CCL5. Blood advances. (2021) 5:4338–51. doi: 10.1182/bloodadvances.2021004203, PMID: 34516642 PMC8579261

[55] Kan Y Ge P Wang X Xiao G Zhao H . SIRT1 rs3758391 polymorphism and risk of diffuse large B cell lymphoma in a Chinese population. Cancer Cell Int. (2018) 18:163. doi: 10.1186/s12935-018-0659-z, PMID: 30377410 PMC6196412

[56] Harris J Lang T Thomas JPW Sukkar MB Nabar NR Kehrl JH . Autophagy and inflammasomes. Mol Immunol. (2017) 86:10–5. doi: 10.1016/j.molimm.2017.02.013, PMID: 28249679

[57] Sadria M Layton AT . Interactions among mTORC, AMPK and SIRT: a computational model for cell energy balance and metabolism. Cell Commun Signal. (2021) 19:57. doi: 10.1186/s12964-021-00706-1, PMID: 34016143 PMC8135154

[58] Forbes SA Bindal N Bamford S Cole C Kok CY Beare D . COSMIC: mining complete cancer genomes in the Catalogue of Somatic Mutations in Cancer. Nucleic Acids Res. (2010) 39:D945–50. doi: 10.1093/nar/gkq929, PMID: 20952405 PMC3013785

[59] Liu GY Sabatini DM . mTOR at the nexus of nutrition, growth, ageing and disease. Nat Rev. (2020) 21:183–203. doi: 10.1038/s41580-019-0199-y, PMID: 31937935 PMC7102936

[60] Saxton RA Sabatini DM . mTOR signaling in growth, metabolism, and disease. Cell. (2017) 168:960–76. doi: 10.1016/j.cell.2017.02.004, PMID: 28283069 PMC5394987

[61] Luo Y Lu S Gao Y Yang K Wu D Xu X . Araloside C attenuates atherosclerosis by modulating macrophage polarization via Sirt1-mediated autophagy. Aging. (2020) 12:1704–24. doi: 10.18632/aging.102708, PMID: 31986489 PMC7053643

[62] Zhang T Fang Z Linghu KG Liu J Gan L Lin L . Small molecule-driven SIRT3-autophagy-mediated NLRP3 inflammasome inhibition ameliorates inflammatory crosstalk between macrophages and adipocytes. Br J Pharmacol. (2020) 177:4645–65. doi: 10.1111/bph.15215, PMID: 32726464 PMC7520450

[63] Vitale I Manic G Coussens LM Kroemer G Galluzzi L . Macrophages and metabolism in the tumor microenvironment. Cell Metab. (2019) 30:36–50. doi: 10.1016/j.cmet.2019.06.001, PMID: 31269428

[64] Lee IH . Mechanisms and disease implications of sirtuin-mediated autophagic regulation. Exp Mol Med. (2019) 51:1–11. doi: 10.1038/s12276-019-0302-7, PMID: 31492861 PMC6802627

[65] Salminen A Kaarniranta K . SIRT1: regulation of longevity via autophagy. Cell signalling. (2009) 21:1356–60. doi: 10.1016/j.cellsig.2009.02.014, PMID: 19249351

[66] Wang Z Guo W Zhang X Wei Y Zhang W Du N . Tumor microenvironment-associated oxidative stress impairs SIRT1 secretion to suppress anti-tumor immune response. Cell Rep. (2025) 44:115679. doi: 10.1016/j.celrep.2025.115679, PMID: 40343797

[67] Lu T Yang J Cai Y Ding M Yu Z Fang X . NCAPD3 promotes diffuse large B-cell lymphoma progression through modulating SIRT1 expression in an H3K9 monomethylation-dependent manner. J advanced Res. (2025) 68:163–78. doi: 10.1016/j.jare.2024.02.024, PMID: 38432395 PMC11785590

[68] Tang Y Ju W Liu Y Deng Q . The role of SIRT1 in autophagy and drug resistance: unveiling new targets and potential biomarkers in cancer therapy. Front Pharmacol. (2024) 15:1469830. doi: 10.3389/fphar.2024.1469830, PMID: 39403142 PMC11471651

[69] Iside C Scafuro M Nebbioso A Altucci L . SIRT1 activation by natural phytochemicals: an overview. Front Pharmacol. (2020) 11:1225. doi: 10.3389/fphar.2020.01225, PMID: 32848804 PMC7426493

[70] Tang X Ding H Liang M Chen X Yan Y Wan N . Curcumin induces ferroptosis in non-small-cell lung cancer via activating autophagy. Thorac cancer. (2021) 12:1219–30. doi: 10.1111/1759-7714.13904, PMID: 33656766 PMC8046146

[71] Lu B Zhang D Wang X Lin D Chen Y Xu X . Targeting SIRT1 to inhibit the proliferation of multiple myeloma cells. Oncol Lett. (2021) 21(4):306. doi: 10.3892/ol.2021.12567, PMID: 33732382 PMC7905587

[72] Alinari L . Toward autophagy-targeted therapy in lymphoma. Blood. (2017) 129:1740–2. doi: 10.1182/blood-2017-02-764639, PMID: 28360356

[73] Braza MS van Leent MMT Lameijer M Sanchez-Gaytan BL Arts RJW Perez-Medina C . Inhibiting inflammation with myeloid cell-specific nanobiologics promotes organ transplant acceptance. Immunity. (2018) 49:819–28.e6. doi: 10.1016/j.immuni.2018.09.008, PMID: 30413362 PMC6251711

[74] Fay F Hansen L Hectors S Sanchez-Gaytan BL Zhao Y Tang J . Investigating the cellular specificity in tumors of a surface-converting nanoparticle by multimodal imaging. Bioconjugate Chem. (2017) 28:1413–21. doi: 10.1021/acs.bioconjchem.7b00086, PMID: 28316241 PMC5567755

[75] Ochando J Braza MS . Nanoparticle-based modulation and monitoring of antigen-presenting cells in organ transplantation. Front Immunol. (2017) 8:1888. doi: 10.3389/fimmu.2017.01888, PMID: 29312352 PMC5743935

[76] Goedhart J Luijsterburg MS . VolcaNoseR is a web app for creating, exploring, labeling and sharing volcano plots. Sci Rep. (2020) 10:20560. doi: 10.1038/s41598-020-76603-3, PMID: 33239692 PMC7689420

