# Supplementary figures and images for: In the diffuse large B-cell lymphoma microenvironment, SIRT1 is upregulated and correlated with a pro-inflammatory macrophage signature and autophagy-related gene expression
Source: Front Immunol. 2026 Feb 4;17:1701514. doi: 10.3389/fimmu.2026.1701514 (PMC12913385; doi:10.3389/fimmu.2026.1701514)

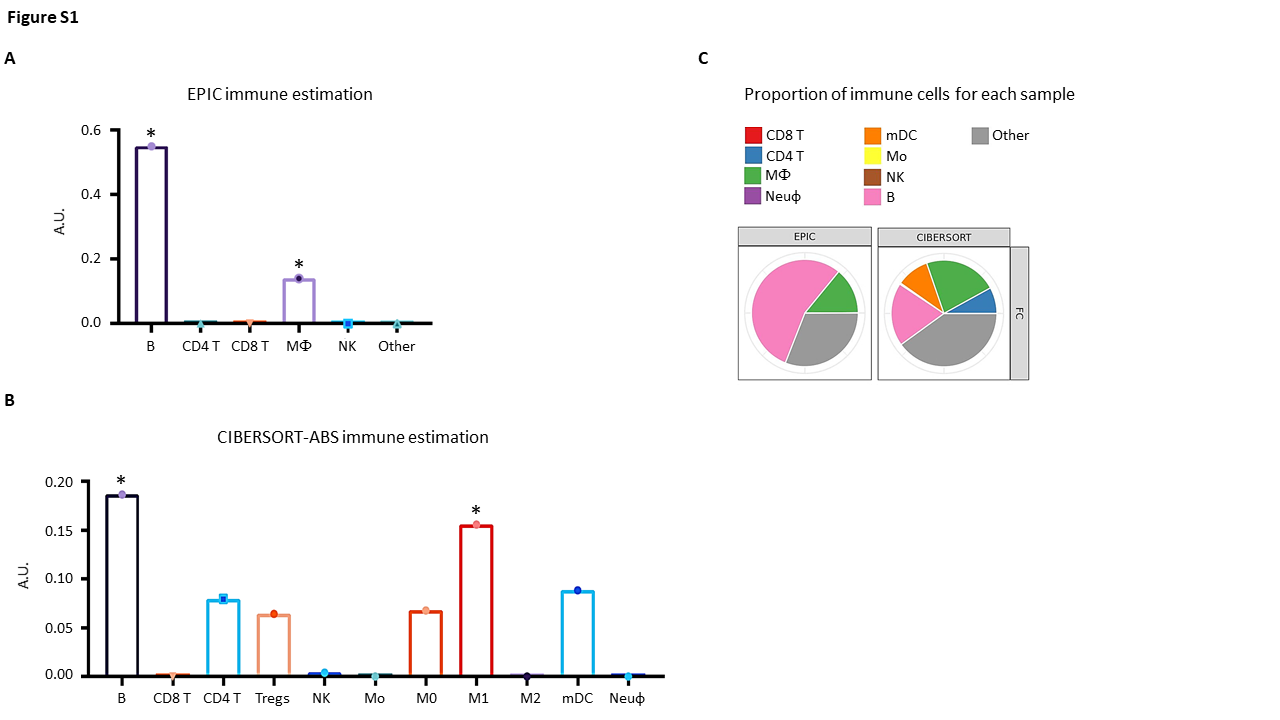

Supplement: Supplementary Figure 1 — The expression of most differentially regulated genes (A) and inflammation/autophagy-related genes (B) was analyzed using the raw transcriptomic data of DLBCL versus spleen samples published in Serna, L et al., 2023 (Supplementary Table S2) and those displayed in this current study (Supplementary Table S3). Volcano plots were generated with VulcaNoseR (76). [file Image1.tif]

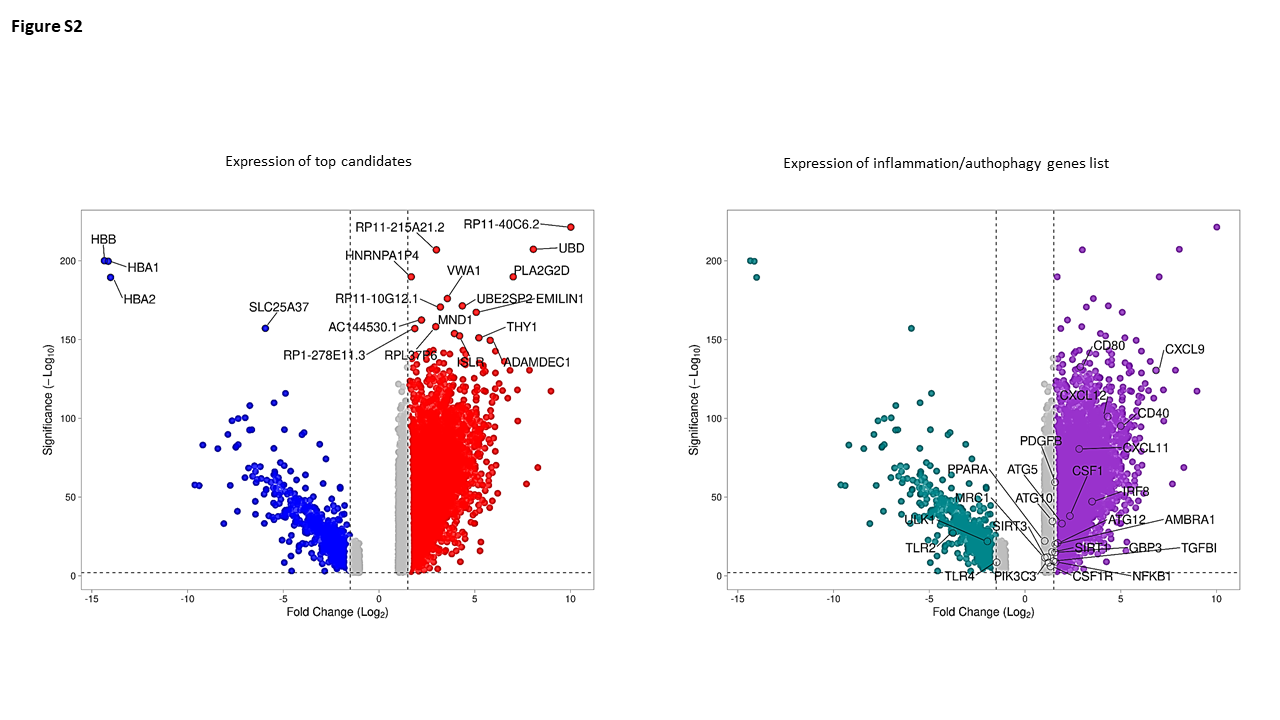

Supplement: Supplementary Figure 2 — Changes in each immune cell type proportion in 47 DLBCL samples (TCGA database) and 337 spleen tissue samples (GTEx database) assessed with the TIMER2.0 tool. (A) Immune infiltration estimation in arbitrary units (A.U.) of B cells (B), CD4+ T cells, CD8+ T cells, macrophages (MФ), natural killer (NK) cells, and other cells using the EPIC algorithm. (B) Immune infiltration estimation (A.U.) of B cells (B), CD8+ T cells, CD4+ T cells, regulatory T cells (Tregs), natural killer (NK) cells, monocytes (Mo), M0, M1 and M2 macrophages, mature dendritic cells (mDC), and neutrophils (Neuφ) using the CYBERSORT algorithm. (C) Proportion (assessed with the TIMER2.0 tool) of CD8+ T cells, CD4+ T cells, macrophages (MФ), neutrophils (Neuφ), mature dendritic cells (mDC), monocytes (Mo), natural killer cells (NK) and B cells (B) using the EPIC and CIBERSORT algorithms. One-way ANOVA was used for the comparison of the different immune cell types and proportions. P ≤0.05 was considered significant. [file Image2.tif]

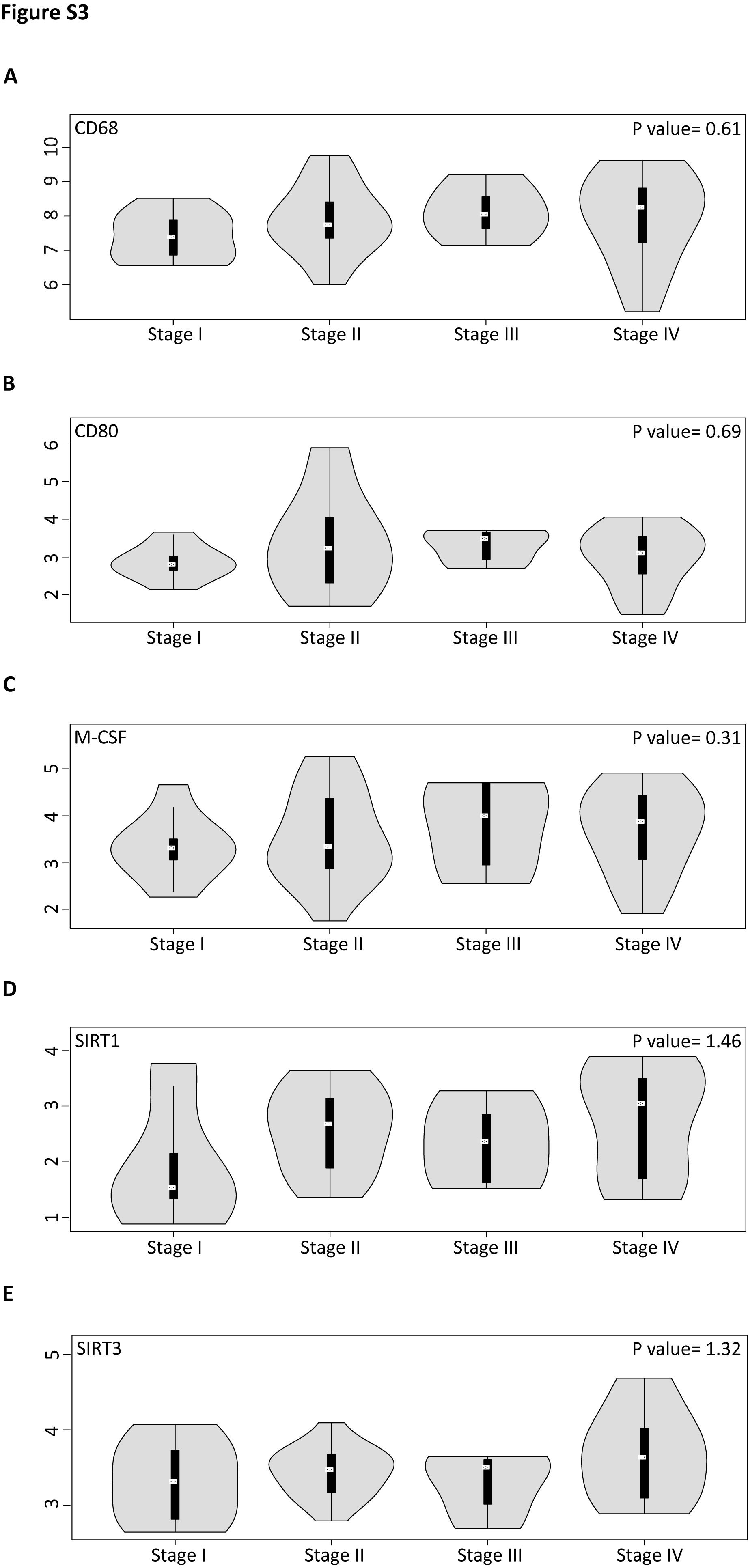

Supplement: Supplementary Figure 3 — Violin plots displaying the expression profiles of CD68 (A), CD80 (B), M-CSF (C), SIRT1 (D) and SIRT3 (E) in DLBCL samples classified according to their stage. [file Image3.tif]

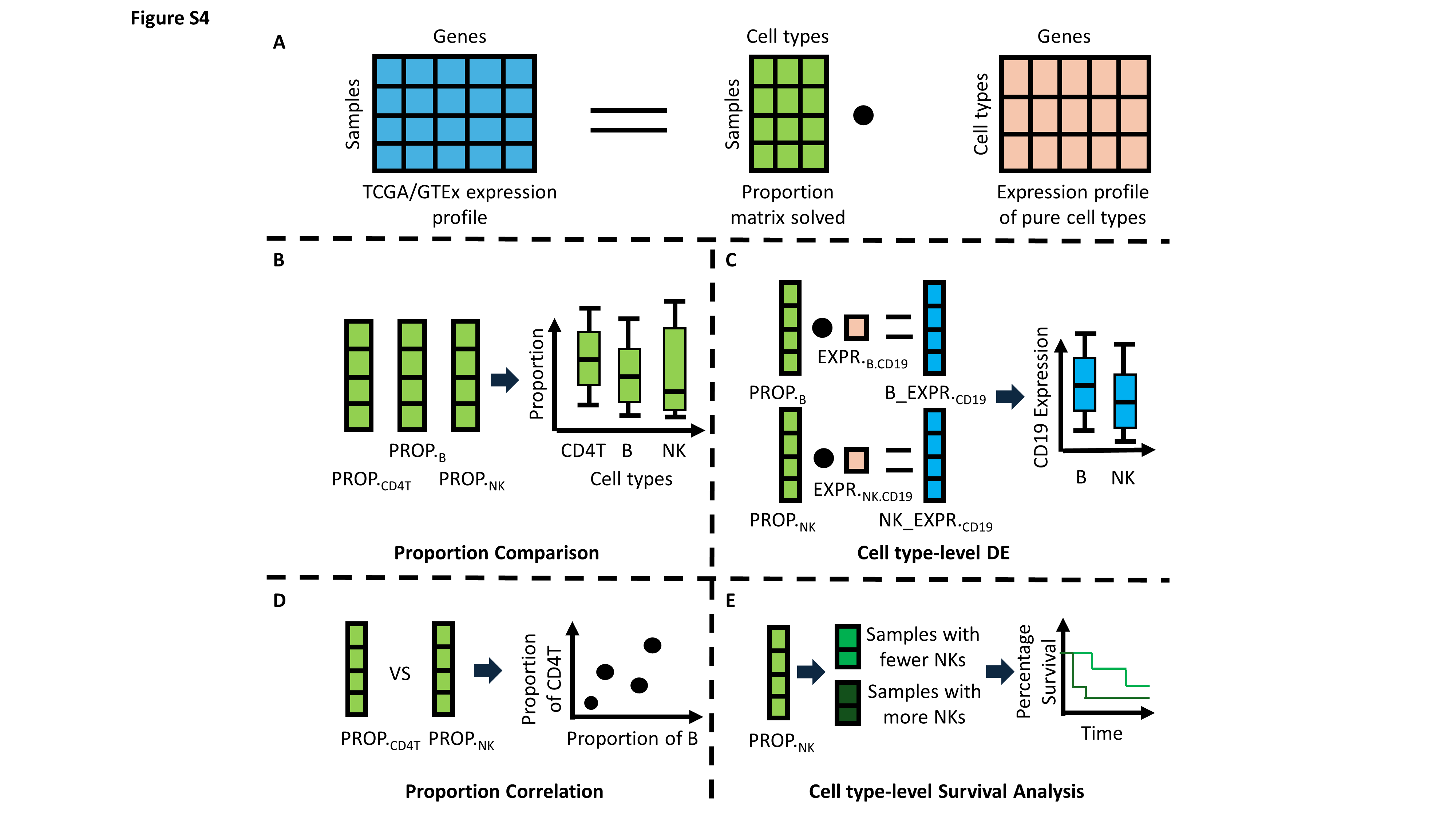

Supplement: Supplementary Figure 4 — Schematic overview of the deconvolution-based analysis in GEPIA2021. (A) Bulk gene expression profiles from TCGA and GTEx datasets are deconvoluted using CIBERSORT, EPIC, or quanTIseq to estimate the cell-type proportion matrix and cell-type-specific expression profiling matrix across samples. (B) Proportion comparison: cell type proportions (e.g., CD4+ T cells, B cells, NK cells) are quantitatively compared across groups or conditions using the ANOVA test. (C) Cell type-level differential expression (DE): gene expression values (e.g., CD19) are inferred in individual cell types (e.g., B vs. NK cells), allowing differential expression testing in a specific cellular context. (D) Proportion correlation: pairwise correlations between cell type proportions (e.g., CD4+ T cells vs. B cells) across samples are visualized and analyzed using Pearson correlations. (E) Cell type-level survival analysis: samples are stratified based on the abundance of a given cell type (e.g., high vs. low NK cell infiltration), and survival outcomes are compared using the Kaplan–Meier analysis and the log-rank test. [file Image4.tif]

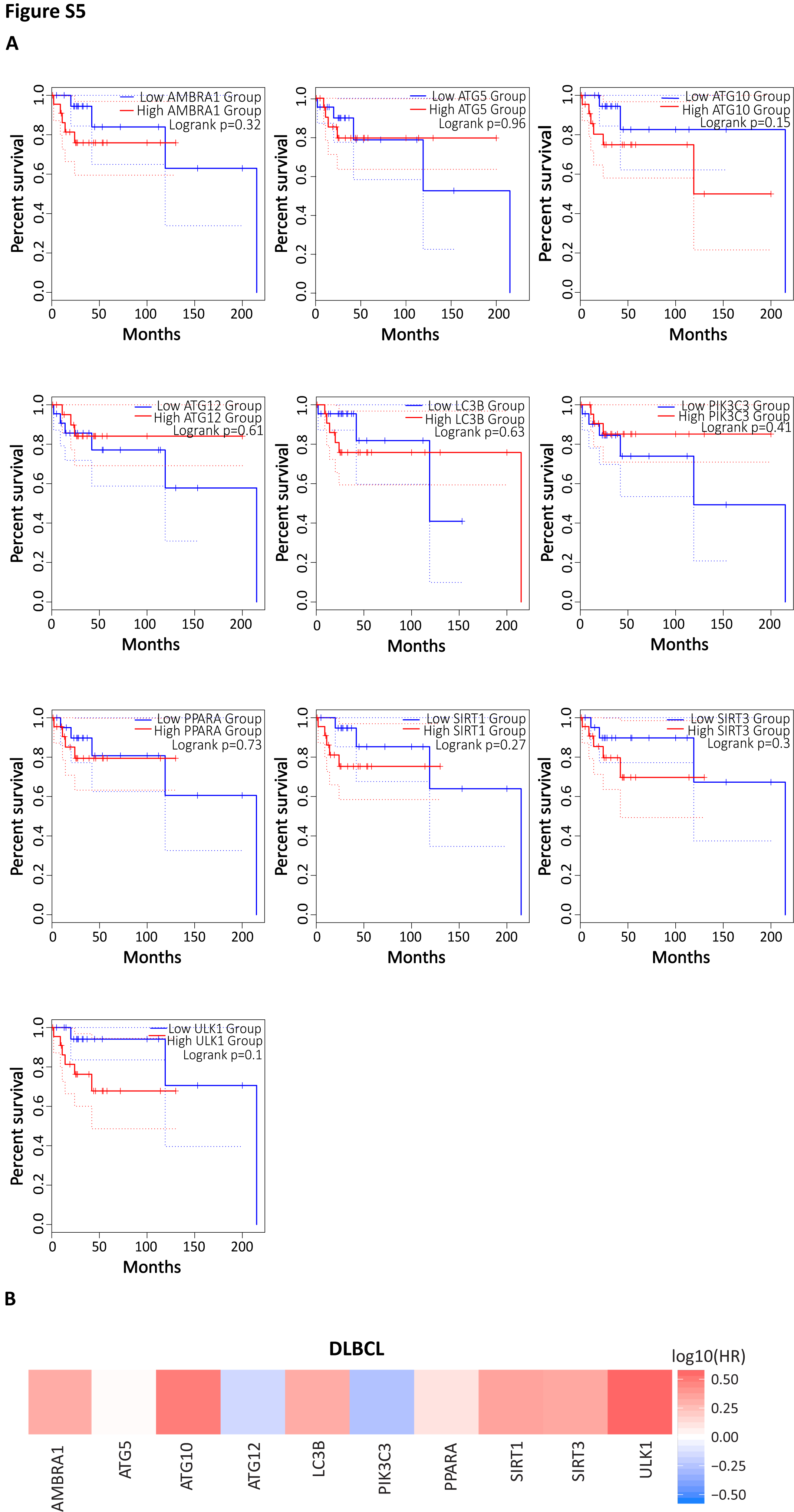

Supplement: Supplementary Figure 5 — (A) Kaplan-Meier survival curve plots based on the expression status of the AMBRA1, ATG5, ATG10, ATG12, LC3B, PIK3C3, PPARA, SIRT1, SIRT3 and ULK1 genes in DLBCL, evaluated in this study (Supplementary Table S3). (B). Survival maps based on the comparison of the survival contribution of the AMBRA1, ATG5, ATG10, ATG12, LC3B, PIK3C3, PPARA, SIRT1, SIRT3 and ULK1 genes, estimated using the Mantel–Cox test. [file Image5.tif]

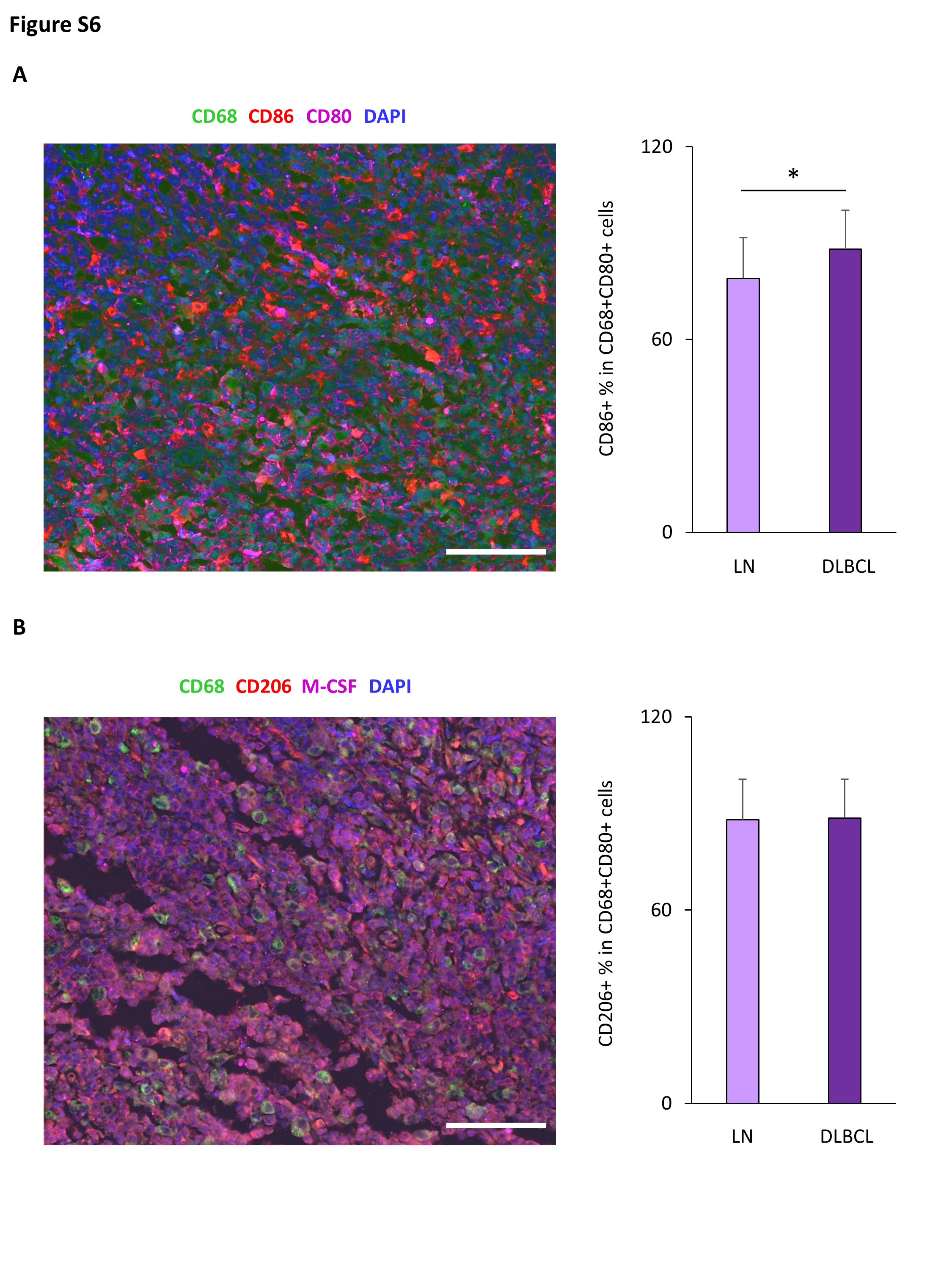

Supplement: Supplementary Figure 6 — Phenotypic validation of M1 and M2 macrophage subtypes in DLBCL and controls. (A) Representative image showing CD68, CD86 and CD86 co-expression in DAPI+ cells in DLBCL samples. Quantitative analysis of CD68+CD86+CD80+ cells in lymph nodes (LN) and DLBCL samples. (B) Representative image showing CD68, CD206 and M-CSF co-expression in DAPI+ cells in DLBCL samples. Quantitative analysis of CD68+CD206+M-CSF+ cells in lymph nodes (LN) and DLBCL samples. Scale bars, 100 µm. * P ≤0.05 was considered significant. [file Image6.tif]
