# Supplementary material for: In the diffuse large B-cell lymphoma microenvironment, SIRT1 is upregulated and correlated with a pro-inflammatory macrophage signature and autophagy-related gene expression
Source: Front Immunol. 2026 Feb 4;17:1701514. doi: 10.3389/fimmu.2026.1701514 (PMC12913385; doi:10.3389/fimmu.2026.1701514)
Supplement: Supplementary Table 3 — Manually curated list of the differentially expressed autophagy-related genes in DLBCL samples and spleen samples with their respective fold change and p values. P ≤0.05 was considered significant. [file DataSheet3.docx]

**Table S3.**

| **Gene Symbol** | **Gene ID** | **Median (Tumor)** | **Median (Normal)** | **Log2**  **(Fold Change)** | **adjp** |
| --- | --- | --- | --- | --- | --- |
| AMBRA1 | ENSG00000110497.14 | 9.240 | 2.420 | 1.582 | 8.72e-21 |
| ATG5 | ENSG00000057663.12 | 9.160 | 1.670 | 1.928 | 6.47e-34 |
| ATG10 | ENSG00000152348.15 | 3.350 | 0.610 | 1.434 | 2.58e-35 |
| ATG12 | ENSG00000145782.12 | 29.270 | 8.290 | 1.704 | 1.29e-21 |
| LC3B | ENSG00000101460.12 | 30.581 | 8.770 | 1.693 | 2.05e-19 |
| PIK3C3 | ENSG00000078142.11 | 27.059 | 10.970 | 1.229 | 3.55e-9 |
| PPARA | ENSG00000186951.16 | 2.300 | 0.620 | 1.026 | 7.14e-23 |
| SIRT1 | ENSG00000096717.11 | 4.530 | 1.080 | 1.411 | 5.74e-16 |
| SIRT3 | ENSG00000142082.14 | 10.340 | 4.100 | 1.153 | 1.20e-12 |
| SIRT6 | \|  \| ENSG00000077463.14 \| \| --- \| --- \| | 28.430 | 9.440 | 1.495 | 2.71e-20 |
| ULK1 | ENSG00000177169.9 | 4.550 | 20.659 | -1.964 | 1.27e-22 |
